# Comparisons of disease cluster patterns, prevalence and health factors in the USA, Canada, England and Ireland

**DOI:** 10.1186/s12889-021-11706-8

**Published:** 2021-09-15

**Authors:** Belinda Hernández, Stacey Voll, Nathan A. Lewis, Cathal McCrory, Arthur White, Lucy Stirland, Rose Anne Kenny, Richard Reilly, Craig P. Hutton, Lauren E. Griffith, Susan A. Kirkland, Graciela Muniz Terrera, Scott M. Hofer

**Affiliations:** 1 The Irish Longitudinal Study on Ageing, Department of Medical Gerontology, School of Medicine, Trinity College, The University of Dublin, Dublin, Ireland; 2 Institute on Aging and Lifelong Health, University of Victoria, Victoria, Canada; 3 Department of Psychology, University of Victoria, Victoria, Canada; 4 School of Computer Science and Statistics, Trinity College, The University of Dublin, Dublin, Ireland; 5 Edinburgh Dementia Prevention and Division of Psychiatry, Centre for Clinical Brain Sciences, University of Edinburgh, Edinburgh, UK; 6 Mercer’s Institute for Successful Ageing, St. James’s Hospital, Trinity College, The University of Dublin, Dublin, Ireland; 7 School of Engineering, Trinity College, The University of Dublin, Dublin, Ireland; 8 Trinity Centre for Biomedical Engineering, Trinity College, The University of Dublin, Dublin, Ireland; 9 Division of Medical Sciences, University of Victoria, Victoria, Canada; 10 Department of Health Research Methods, Evidence, and Impact, McMaster University, Hamilton, Ontario Canada; 11 Department of Community Health &Epidemiology and Medicine, Dalhousie University, Halifax, Nova Scotia Canada

**Keywords:** Multimorbidity, Disease clusters, Latent class analysis, Ageing studies

## Abstract

**Background:**

Identification of those who are most at risk of developing specific patterns of disease across different populations is required for directing public health policy. Here, we contrast prevalence and patterns of cross-national disease incidence, co-occurrence and related risk factors across population samples from the U.S., Canada, England and Ireland.

**Methods:**

Participants (*n* = 62,111) were drawn from the US Health and Retirement Study (*n* = 10,858); the Canadian Longitudinal Study on Ageing (*n* = 36,647); the English Longitudinal Study of Ageing (*n* = 7938) and The Irish Longitudinal Study on Ageing (*n* = 6668). Self-reported lifetime prevalence of 10 medical conditions, predominant clusters of multimorbidity and their specific risk factors were compared across countries using latent class analysis.

**Results:**

The U.S. had significantly higher prevalence of multimorbid disease patterns and nearly all diseases when compared to the three other countries, even after adjusting for age, sex, BMI, income, employment status, education, alcohol consumption and smoking history. For the U.S. the most at-risk group were younger on average compared to Canada, England and Ireland. Socioeconomic gradients for specific disease combinations were more pronounced for the U.S., Canada and England than they were for Ireland. The rates of obesity trends over the last 50 years align with the prevalence of eight of the 10 diseases examined. While patterns of disease clusters and the risk factors related to each of the disease clusters were similar, the probabilities of the diseases within each cluster differed across countries.

**Conclusions:**

This information can be used to better understand the complex nature of multimorbidity and identify appropriate prevention and management strategies for treating multimorbidity across countries.

**Supplementary Information:**

The online version contains supplementary material available at 10.1186/s12889-021-11706-8.

## Introduction

Multimorbidity (the presence of two or more medical conditions simultaneously [1–3]) is an increasingly important topic as there is growing evidence that multimorbidity is now the norm rather than the exception in ageing populations [1, 4–6]. Multimorbidity is strongly associated with physical and functional decline, mortality [4, 5, 7] decreased quality of life [8, 9] and increased health care usage and costs [4, 7, 10]. The Academy of Medical Sciences identified the investigation of disease clusters and their corresponding risk factors as a critical gap in our understanding of multimorbidity [3].

Our aim is to provide a cross-country comparison of disease prevalence as well as the unique patterns of multimorbidity as disease clusters and associated risk factors to uncover how differences in demographics, socio-economic status and health behaviors affect the combinations of diseases within and across four countries: United States, Canada, England and Ireland. Selection of these four countries (all ranked globally in the top 14 according to the 2018 United Nations human development index) allows for comparison across the range of public healthcare delivery systems of North America and Europe.

An abundant body of literature compares health outcomes between the U.S. and other developed countries; with several publications reporting health disadvantage and higher disease prevalence for the U.S. [11–16]. In particular, Banks et al. showed that the U.S. population when compared to England’s population had worse health and higher prevalence for seven common diseases regardless of level of socio-economic status, demographics and behavioral risk factors [12]. Although the U.S., Canada, England and Ireland all have some form of publicly funded health care for those aged over 65, the level of public care offered varies over countries. England and Canada have universal healthcare for all ages; Ireland has a mixed public and private healthcare system, with public health care for those below an identified income level, and a range of community and hospital services free of charge for all, despite income levels. In contrast, the United States has a mostly privatized system.

The United States, despite having a per capita expenditure on health care that is 1.9–2.8 times higher than Canada, Ireland or England, has the lowest life expectancy, highest mortality rate and highest number of disability-adjusted life years lost due to non-communicable and largely preventable diseases of these four countries (Additional file 1: Table 1).

Evidence required to address these health policy issues requires an understanding of the complexities of multimorbidity and related health factors. Here we have the opportunity to do so across multiple countries, yielding findings of the common trends of health and the specific patterns of multimorbidity unique to the U. S, Canada, Ireland and England.

## Methods

Analysis was based on cross-sectional data from a total of 62,111 respondents aged 52–85, participating in the 2012 (wave 11) U.S. Health and Retirement Survey (HRS) (*n* = 10,858) [17]; 2012–2013 (wave 6) English Longitudinal Study on Ageing (ELSA) (*n* = 7938) [18]; 2012 (wave 2) The Irish Longitudinal Study in Ageing (TILDA) (*n* = 6668) [19] and 2010–2015 baseline of the Canadian Longitudinal Study on Aging (CLSA) (*n* = 36,647) [20]. The design of these studies has been comprehensively described elsewhere [17–21] but for completeness is explained in Additional file 2 Section 1.

To eliminate differences in disease prevalence and patterns due to the disparate racial structures, analysis was limited to the non-Hispanic white subpopulation. A breakdown of the cohort characteristics for all four countries can be seen in Additional file 3: Tables 1–4.

### Self-reported diagnoses and risk factors

Nine self-reported medical conditions were identified as common across all four studies: hypertension, diabetes, stroke (including transient ischemic attack), angina, myocardial infarction (MI), arthritis, cancer (not including minor skin cancers), lung disease (at least one of: emphysema, chronic bronchitis or chronic obstructive pulmonary disease) and osteoporosis. A tenth condition included psychological disorders of anxiety/mood (Psych 1) (CLSA, HRS) and/or psychiatric problems (Psych 2) (TILDA, HRS, ELSA).

### Covariates

Covariates included age, sex and socioeconomic status (SES), characterized by education level and household income tertiles. Employment status was also included to ensure differences in household income were more reflective of permanent features of SES and not confounded by a lack of income due to temporary unemployment or retirement. Health factors controlled for were body mass index, smoking history and alcohol consumption. For detailed information on the covariates and harmonization of medical conditions across studies, see Additional file 2 Section 2.

### Statistical analysis

Cross-sectional survey weights were used to report population representative disease prevalence using STATA 15. Crude population prevalence of disease was calculated using the tab command in STATA 15. Odds ratios for disease presence and risk factors were calculated using a survey-weighted logistic regression for each disease. This was implemented with the svy:logit command in STATA15. When making comparisons directly to the U.S., we pooled data across countries, ensuring cluster and strata variables across countries were accounted for; country-level weights were scaled to have a common mean and standard deviation 1 to prevent countries with weights on a larger scale dominating the analysis. Fully adjusted income, education and BMI gradients for each disease were identified and calculated with the addition of an interaction term by country. The marginal effect of each respective variable was then extracted assuming all other confounding variables were equal. This was performed using the margins command in STATA15.

Disease patterns were identified using Latent Class Analyses (LCA) which were population weighted in all cases and took into account the stratification and clustering inherent in the cohort sampling designs. LCA is a model-based clustering method for multivariate categorical data and has previously been applied in the analysis of multimorbidity [22, 23]. In the case of multimorbidity, clustering using LCA is more appropriate than standard distance-based methods, such as k-means or hierarchical clustering, since the appropriate probability distribution for the data is readily available. Furthermore, LCA allows extra flexibility for diseases to have partial membership across multiple clusters unlike other more limiting distance-based clustering methods.

Two sets of parameters underlie the model: the group probability *τ* and item probability *θ*. The group probability parameter represents the a priori probability that an observation belongs to a particular group, so that P(Group g) = τ_*g*_. The item response probability represents the probability of a success for a given item, conditional on group membership, so that P(Item m = 1 | Group g) = θ_*gm*_.

More formally, let *X* = *X*_1_, …, *X*_*n*_ denote M-dimensional vector-valued binary random variables, composed of G groups. The observed-data likelihood distribution for the data *X* can then be written: $p\Big(X\left|\ \uptheta, \uptau \right)={\prod}_{\mathrm{i}=1}^{\mathrm{n}}{\sum}_{\mathrm{g}=1}^{\mathrm{G}}{\uptau}_{\mathrm{g}}{\prod}_{\mathrm{m}=1}^{\mathrm{M}}{\uptheta}_{\mathrm{g}\mathrm{m}}^{{\mathrm{x}}_{\mathrm{i}\mathrm{m}}}{\left(1-{\uptheta}_{\mathrm{g}\mathrm{m}}\right)}^{1-{\mathrm{X}}_{\mathrm{i}\mathrm{m}}}$.

The naïve Bayes assumption that observations are conditionally independent based on group membership has been made for this model. Direct inference using the observed-data likelihood is typically difficult and is facilitated by the introduction of latent variables Z = Z_1_, …, Z_*n*_. Each Z_*i*_ = Z_*i*1_, …Z_*iG*_ is a G-dimensional vector, representing the true cluster membership of *X*_*i*_ as a multinomial random variable. That is, suppose that the true group membership is known for each X_*i*_ and is denoted by Z_*ig*_ = 1 if observation *i* belongs to Group *g*, otherwise Z_*ig*_ = 0. The complete-data density for an observation (X_*i*_, Z_*i*_) is then $\mathrm{p}\left(\mathrm{X},\mathrm{Z}|\uptheta, \uptau \right)={\prod}_{\mathrm{i}=1}^{\mathrm{n}}{\prod}_{\mathrm{g}=1}^{\mathrm{G}}{\left\{{\uptau}_{\mathrm{g}}{\prod}_{\mathrm{m}=1}^{\mathrm{M}}{\uptheta}_{\mathrm{g}\mathrm{m}}^{{\mathrm{x}}_{\mathrm{i}\mathrm{m}}}{\left(1-{\uptheta}_{\mathrm{g}\mathrm{m}}\right)}^{1-{\mathrm{X}}_{\mathrm{i}\mathrm{m}}}\right\}}^{{\mathrm{Z}}_{\mathrm{i}\mathrm{g}}}.$ LCA thus allows the data to be summarised at a global and local level. The parameters *θ* and *τ* summarise the overall behaviour of the clusters in the data, while each variable *Z*_*i*_ informs us of the cluster membership, and thus behaviour, of an individual observation *i*.

Inference for our LCA models was performed using an expectation-maximisation (EM) algorithm. This works in two steps: the E-step, where *Z* is estimated, based on the current values of *θ* and *τ*, and the M-step, where the complete data likelihood is maximised with respect to *θ* and *τ* based on the current value of *Z*. The algorithm proceeds iteratively until it has deemed to converge; that is, once parameter estimates remain more or less unchanged after successive iterations.

As the true number of groups *G* is not known in advance, each LCA model was run over a range of 1–10 groups. The number of clusters was then chosen using the Bayesian information criterion (BIC), where $BIC=-2\log \mathrm{p}\left(\mathrm{X}\ \right|\uptheta, \tau \Big)+\left( GM+G-1\right)\log \left(\sum \limits_{\mathrm{i}=1}^{\mathrm{n}}{w}_i\right)$; *w*_*i*_ is the survey weight attached to observation *i* and logp(X |θ, τ) is the survey weighted pseudo-loglikelihood. Here a lower value of BIC indicates a more suitable choice of model. In many practical examples as was performed in this current work a balance has to be found between model parsimony and model fit and so an “elbow” is usually identified whereby the addition of clusters has diminishing returns to model fit improvement. We applied LCA using the software package lcca in R [24]. Code to implement this analysis and BIC values for all models assessed are provided in Additional file 4.

## Results

### Individual disease prevalence

**Fig. 1:**
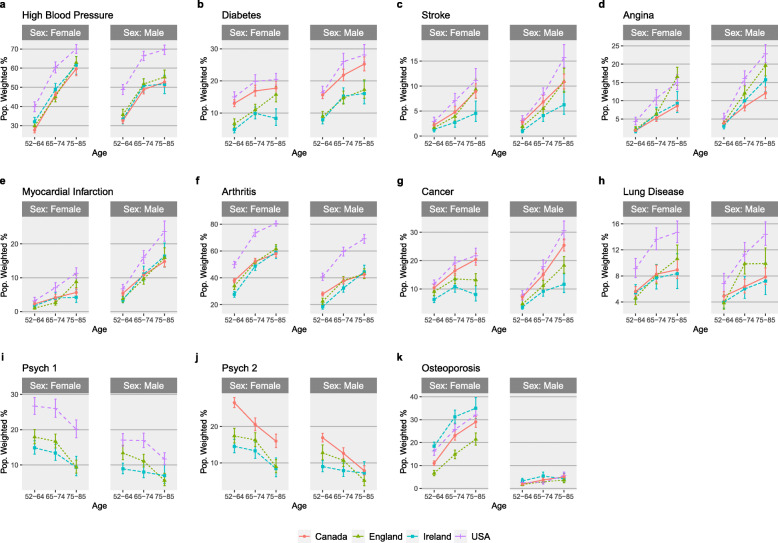
Crude disease prevalence (weighted %) stratified across sex and age groups for the four countries

Figure 1 shows the crude population weighted prevalence of the 10 medical conditions by sex and age categories. Table 1 shows the odds ratio of each condition compared to the U.S. after adjusting for confounding variables. Here, it can be seen, that the U.S. had significantly higher prevalence than England for all 10 medical conditions and for all, except diabetes, when compared to Canada, even in adjusted models. The U.S. had significantly higher prevalence for all, except osteoporosis, when compared to Ireland.

**Table 1:** Odds ratios and standard errors for each medical condition compared to the U.S. as reference

	IRELAND	ENGLAND	CANADA
**Hypertension**	0.60 (0.26) ‡	0.54 (0.02) ‡	0.53 (0.02) ‡
**Diabetes**	0.46 (0.03) ‡	0.54 (0.03) ‡	1.06 (0.03)
**Stroke**	0.36 (0.04) ‡	0.61 (0.05) †	0.84 (0.04) *
**Angina**	0.58 (0.05) †	0.76(0.05) *	0.53 (0.03) ‡
**Myocardial Infarction**	0.54 (0.04) †	0.55 (0.04) †	0.67 (0.03) ‡
**Arthritis**	0.36 (0.01) ‡	0.44 (0.02) ‡	0.46 (0.01) ‡
**Cancer**	0.45 (0.03)‡	0.57 (0.03)‡	0.84 (0.03) †
**Lung Disease**	0.52 (0.04) †	0.58 (0.04) †	0.61 (0.03)‡
**Psychiatric Problems (Psych2)**	0.44 (0.03) ‡	0.59 (0.03) ‡	NA
**Osteoporosis**	1.26 (0.07) *	0.51 (0.03) †	0.72 (0.03) †

*p-value<0.001, †*p*-value<0.00001, ‡*p*-value<1e-16. Note. Odds ratios have controlled for age, sex, education, income, employment status, smoking history, BMI and alcohol consumption frequency. Odds ratios also take account of survey design and weighting in all cases

The U.S. and Canada had very similar prevalence of diabetes, regardless of age and sex (Fig. 1). The odds of having diabetes in the U.S. was approximately double that of Ireland or England (Table 1) even in adjusted models. The U.S. had a pronounced higher prevalence of hypertension, arthritis, and psychological conditions across all age and sex categories. In particular, 56.8% of the U.S. population had arthritis; significantly, higher than the other countries whose prevalence was 32.3–38.2%. Ireland had the highest prevalence of osteoporosis (13.85%) followed closely by the U.S. (13.0%) then Canada (9.8%) and England (7.5%).

Additional files 5, 6 and 7 show the fully adjusted disease prevalence across countries with respect to income, education and BMI. Here it can be seen that income and education gradients are more pronounced for the U.S., especially for lung disease, stroke (with respect to income), myocardial infarction, psychological illnesses (with respect to income), and high blood pressure. The prevalence of psychological illnesses in all cases was inversely related to income, however the opposite was true of education level. Hence those with lower income but who are higher educated are more likely to be diagnosed with a psychological illnesses. In the U.S., 60.7% of adults, aged 52–85, had two or more medical conditions. This is considerably higher than the other countries: Canada 45.3%, England 42.1% and Ireland 38.6%.

### Disease cluster compositions

**Fig. 2:**
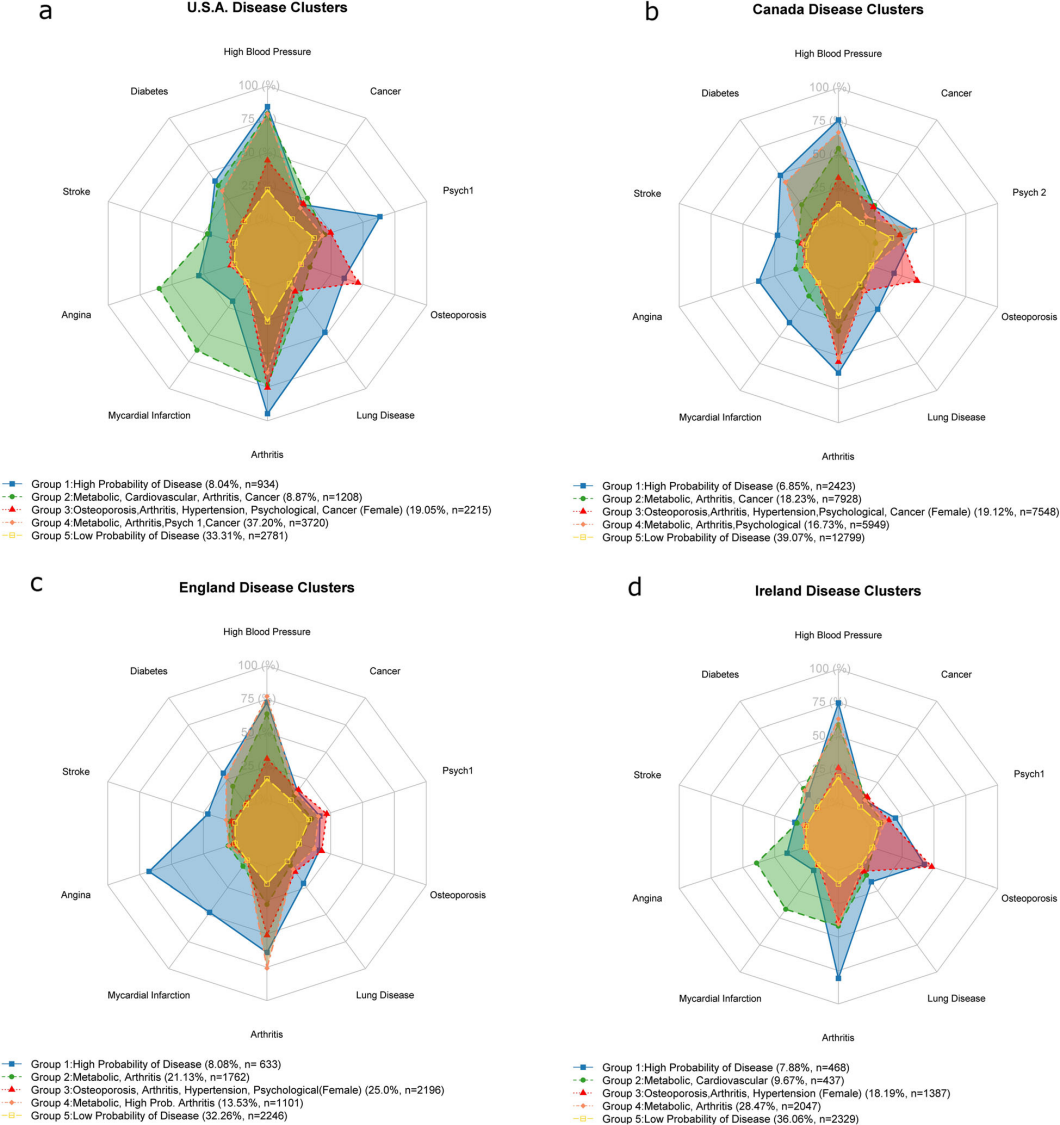
Item response probabilities Item Response Probabilities for identified disease clusters for the USA (**a** top left), Canada (**b** top right), England (**c** bottom left) and Ireland (**d** bottom right). Note: Definition of stroke includes transient ischemic attack; lung disease included a self-reported diagnosis or at least one of the following: emphysema, chronic bronchitis or chronic obstructive pulmonary disease; Psych1 was measured as self-reported diagnosis of any emotional, mental or psychological disorder; Psych2 included a self-reported diagnosis or at least one of: depression, anxiety disorder or bipolar disorder; Cancer excluded minor skin cancers and melanoma

**Fig. 3:**
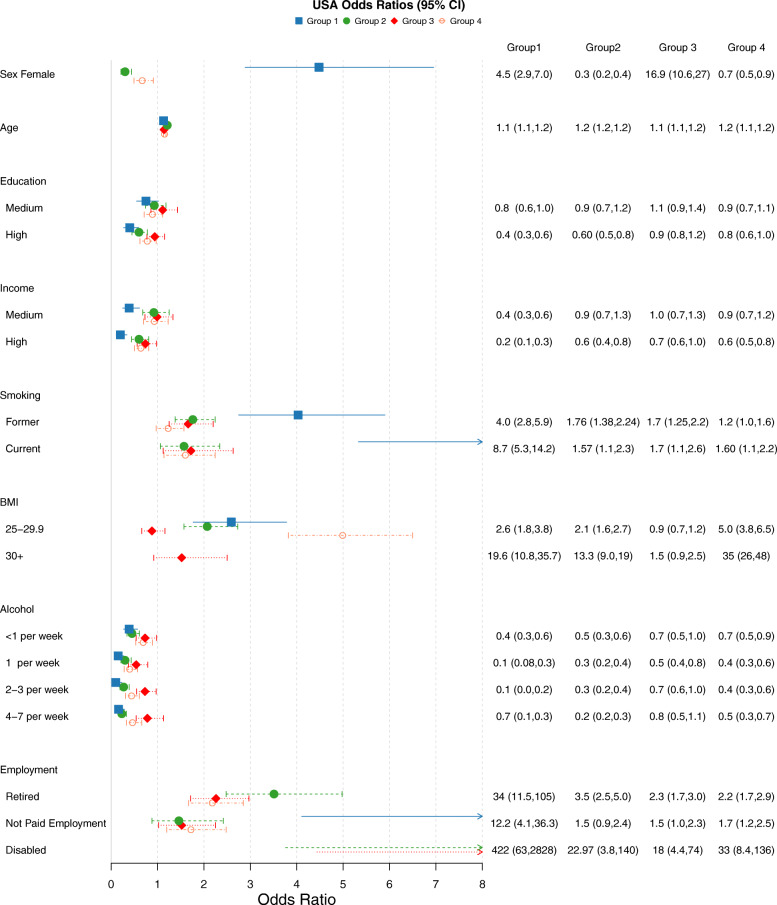
Odds Ratios and 95% confidence intervals of risk factors for each latent class compared to reference “Low probability of disease” class of the HRS sample. Group 1:High Probability of Disease (population weighted prevalence 8.04%, *n* = 934); Group 2:Metabolic, Cardiovascular, Arthritis, Cancer (population weighted prevalence 8.87%, *n* = 1208); Group 3:Osteoporosis,Arthritis, Hypertension, Psychological, Cancer (Female) (population weighted prevalence 19.05%, *n* = 2215)”; Group 4:Metabolic, Arthritis, Psychological, Cancer (population weighted prevalence 37.20%, *n* = 3720)

**Fig. 4:**
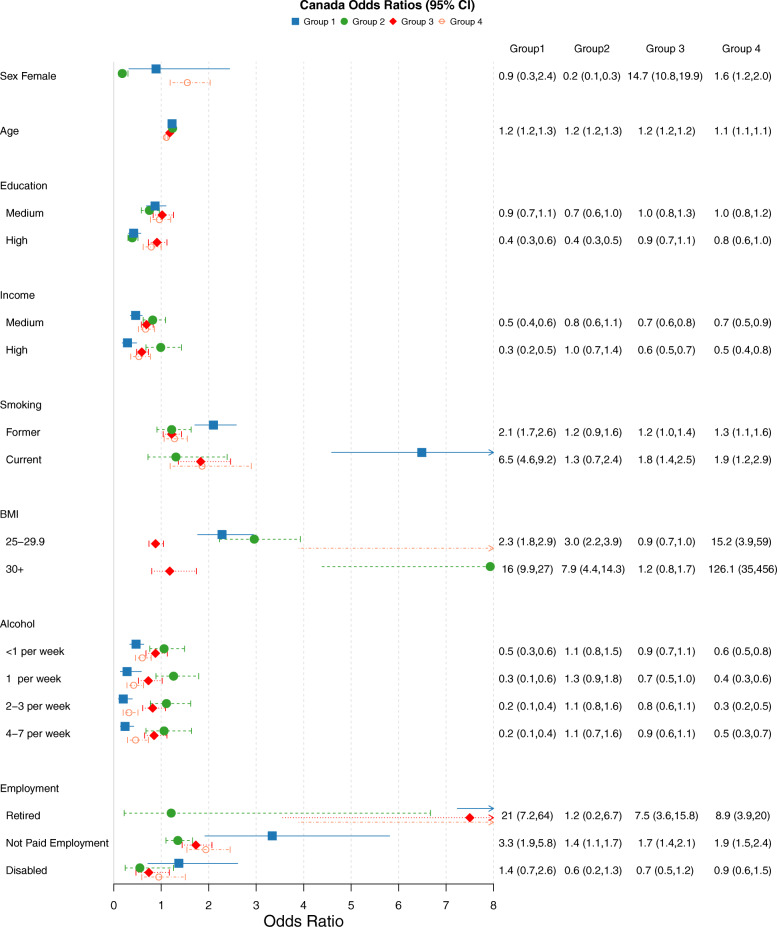
Odds Ratios and 95% confidence intervals of risk factors for each latent class compared to reference “Low probability of disease” class of the CLSA sample. Group 1:High Probability of Disease (population weighted prevalence 6.85%, *n* = 2423); Group 2:Metabolic, Arthritis, Cancer (population weighted prevalence 18.23%, *n* = 7928)”; Group 3:Osteoporosis,Arthritis, Hypertension,Psychological, Cancer (Female) (population weighted prevalence 19.12%, *n* = 7548); Group 4:Metabolic, Arthritis, Psychological (population weighted prevalence 16.73%, *n* = 5949)

**Fig. 5:**
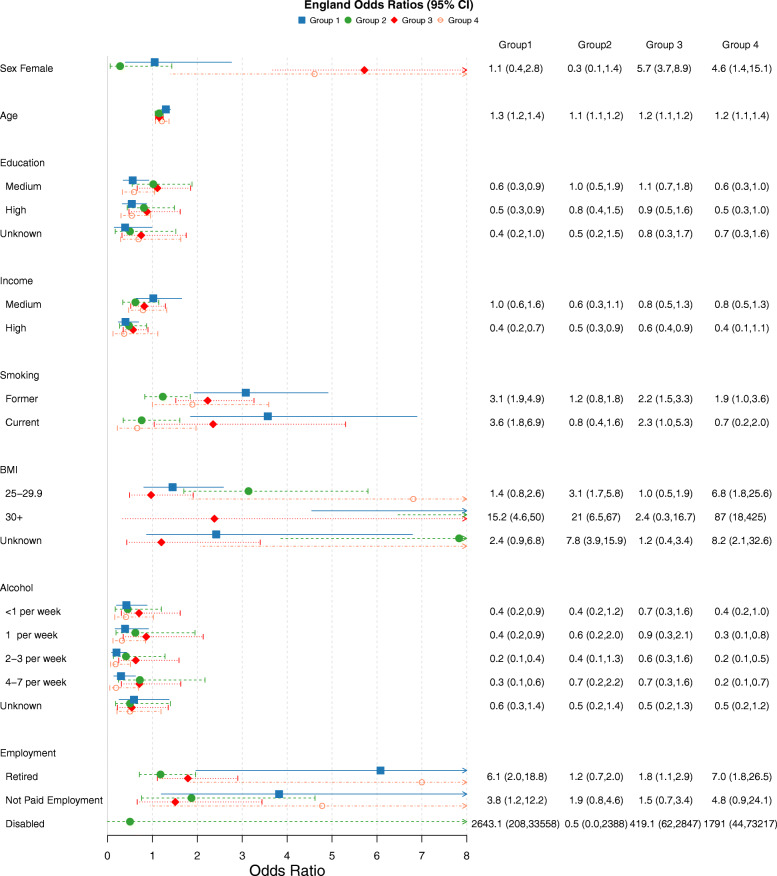
Odds Ratios and 95% confidence intervals of risk factors for each latent class compared to reference “Low probability of disease” class of the ELSA sample. Group 1:High Probability of Disease (population weighted prevalence 8.08%, *n* = 633); Group 2:Metabolic, Arthritis (population weighted prevalence 21.13%, *n* = 1762); Group 3:Osteoporosis, Arthritis, Hypertension, Psychological (Female) (population weighted prevalence 25.0%, *n* = 2196); Group 4:Metabolic, High Prob. Arthritis (population weighted prevalence 13.53%, *n* = 1101)

**Fig. 6:**
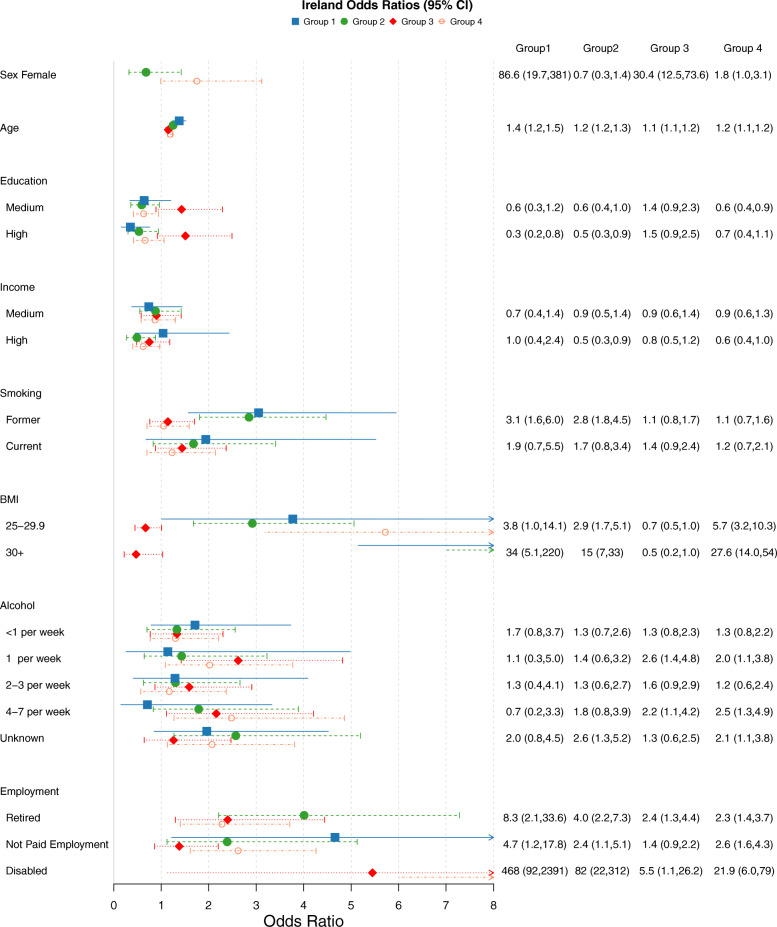
Odds Ratios and 95% confidence intervals of risk factors for each latent class compared to reference “Low probability of disease” class of the TILDA sample. Group 1:High Probability of Disease (population weighted prevalence 7.88%, *n* = 468); Group 2:Metabolic, Cardiovascular (population weighted prevalence 9.67%, *n* = 437); Group 3:Osteoporosis,Arthritis, Hypertension (Female) (population weighted prevalence 18.19%, *n* = 1387); Group 4:Metabolic, Arthritis (population weighted prevalence 28.47%, *n* = 2047)

Five latent classes (disease clusters) were identified for all four cohorts. The item response probabilities for the clusters of each country can be seen in Fig. 2. Figures 3, 4, 5 and 6 show the odds ratios and 95% confidence intervals for risk factors associated with each specific disease pattern compared to the “low probability of disease” group for the U.S., Canada, England and Ireland respectively. The population weighted proportion of each disease pattern for the U.S., Canada, England and Ireland with respect to its risk factors can be seen in Additional files 8, 9, 10 and 11 respectively. In general, the risk factors and their direction of association for disease clusters are consistent across countries (Fig. 3, 4, 5 and 6). However, the composition of clusters across countries varies. For example, the “high probability of disease” group across all countries was associated with higher odds of being older, lower educated, lower income having a smoking history and being obese (Fig. 3, 4, 5 and 6). Regarding the composition of this “high probability of disease” group however, Canada and England had higher probability of myocardial infarction (47.9% England, 37.2% Canada, 6.32% Ireland 19.2% U.S.) and angina (67.6% England, 37.5% Canada, 15.3% Ireland, 29.9% U.S.) (see Group 1 Fig. 2a, b, c, d). For Ireland and the U.S., cardiovascular diseases were separated into a distinct cluster along with high blood pressure, diabetes and arthritis (see Fig. 2 Group 2 a, d).

### BMI

Having an elevated BMI (> 25) was strongly associated with the “high probability of disease” groups across all four countries (see Group 1 Figs. 3, 4, 5 and 6). It was also associated with the two groups which were predominantly cardiometabolic and metabolic in nature across countries: Group 2 and Group 4 (see Fig. 2 (cluster patterns), Figs. 3, 4, 5 and 6 (odds ratios and 95% CI), see also Additional files 8, 9, 10 and 11). Although, older age increased the odds of all disease groups, the “high probability of disease” group for the U.S. had a high proportion of 52–64 year olds of 37.4%. The disease cluster with the highest disease burden for the other countries (Group 1) had a lower proportion of younger participants (31.6% Canada, 14.2% England, 11.4% Ireland).

### Education

The gradient and significance of socioeconomic indicators varied across countries for their respective disease patterns. In general, groups which consisted of high blood pressure, diabetes and arthritis and/or cardiovascular diseases were negatively associated with education. High education was associated with decreased odds of three disease groups for the U.S. and Canada (see Groups 1, 2, 4 Fig. 3 U.S. and Groups 1,2,4 Fig. 4 Canada). High education was also associated with two groups for England (Groups 1, 4 Fig. 5) and Ireland (Groups 1,2 Fig. 6). Across all four countries the disease cluster which predominantly consisted of high blood pressure, arthritis, osteoporosis and in some cases mental illnesses was not associated with education (Group 3 Fig. 2 and Fig. 3, 4, 5 and 6).

### Income

Canada and the U.S. had more pronounced income gradients with regards to disease patterns. For Canada, medium and high income were negatively associated with three clusters (Groups 1,3,4 Fig. 2b and Fig. 4 see also Additional file 9). For the U.S. high income significantly decreased the odds of all four clusters (Figs. 2, 3 and Additional file 8). High income was associated with decreased odds of three groups for England (Groups 1,2,3 Fig. 2c and Fig. 5 see also Additional file 10). Ireland had the least pronounced income gradient and high income was negatively associated with only two groups, both of which had high probability of high blood pressure, diabetes and arthritis and cardiovascular diseases (Groups 2 and 4 Fig. 2d and Fig. 6, see also Additional file 11).

### Smoking

With regards to lifestyle factors, in general having a history of smoking increased the odds of disease for the majority of disease groups. Canada and the U.S. had the most pronounced effect for smoking history where being a current smoker was positively associated with all four disease patterns for the U.S. (Fig. 2a and 3) and all but Group 2 for Canada which was predominantly metabolic, arthritis and cancer (see Group 2 Fig. 2b and Fig. 4). A similar trend was found for being a past smoker. Ireland had the least pronounced effect where being a past smoker was only associated with Group 1 “high probability of disease” and Group 2 which had high prevalence of metabolic and cardiovascular conditions (Groups 1 and 2 Fig. 2d and Fig. 6; see also Additional file 11).

### Alcohol

Alcohol consumption was negatively associated with all disease clusters for the U.S. (Fig. 3) and for two clusters for Canada and England (Canada Group 1: High probability of disease and Group 4: Metabolic, Arthritis, Psychological groups Fig. 2b and Fig. 4; England Group 1: High probability of disease and Group 4: Metabolic, High Probability of Arthritis see Fig. 2c and Fig. 5). Ireland displayed the opposite trend, with alcohol consumption being positively associated with Group 3: Osteoporosis,Arthritis, Hypertension (Female) (population weighted prevalence 18.19%, *n* = 1387) and Group 4: Metabolic, Arthritis see Fig. 2d and Fig. 6.

## Discussion

Overall, the U.S. had significantly higher prevalence of nearly all medical conditions compared to Canada, England and Ireland. This trend persisted even after controlling for age, sex, SES, and health behaviors. This study focused solely on the non-Hispanic white sub-populations of each country to control for the disparities of health outcomes in racial structures of the U.S. and other countries.

### Differences in disease prevalence

The U.S. and Ireland had marked higher prevalence of osteoporosis when compared to England. Differences in genetic susceptibility or vitamin D levels are unlikely to account for such a difference as Ireland and England have similar latitude and a very homogenous genetic structure [25]. A possible explanation lies in increased detection in the U.S. and Ireland as both countries have more of a culture of privatized healthcare and offer affordable scans to diagnose osteoporosis privately in both countries ($85–305 U.S., €80–100 Ireland). In the case of Ireland this theory is further supported by the fact that those with high income and high education (i.e. those who are more likely to have private health insurance and or more disposable income to afford doctor’s fees) had the highest probability of being diagnosed with osteoporosis.

The prevalence of diabetes and cancer were considerably higher in the U.S. and Canada. All four countries offer public screening programs for cervical, breast and bowel cancer so increased detection is unlikely to explain such discrepancies. Ireland and England have a higher cancer mortality rate [26–29] which may partially explain this difference. There were also pronounced geographical similarities in the prevalence of psychological conditions. The U.S. and Canada had much higher prevalence of psychological conditions than Ireland or England. This is likely at least partially due to differences in how psychological conditions are defined and diagnosed across countries. Practitioners in the U.S. and Canada use the Diagnostic and Statistical Manual of Mental Disorders to inform diagnoses whereas England and Ireland use the International Classification of Diseases and Related Health Problems. Previous literature has noted discrepancies in disease classification between these criteria [30–32]. The U.K. and Ireland also have a higher number of psychiatrists working in the mental health sector per 100,000 population (18.9 England, 17.4 Ireland, 14.7 U.S., 10.5 Canada) [33, 34] a higher number of mental health hospitals per 100,000 population (Ireland 0.62, U.S. 0.19, Canada 0.06) and higher number of mental health outpatient facilities per 100,000 population (Ireland 3.83, U.S. 0.37, Canada 0.33) [31–34]. The level of funding for mental health services has a nearly exact inverse relationship to the prevalence of psychological conditions. Although speculative, it may also be that more availability and early access to mental health services in England and Ireland is partially offsetting the onset of chronic mental health conditions in these countries [32, 35].

### Disease patterns

Regarding the analysis of disease clusters and their associated risk factors, all four countries uncovered five similar groups. Knowing disease combinations for a given country can bring about a better understanding of the complex nature of multimorbidity. For example, in all four countries hypertension and arthritis were highly prevalent in three disease clusters. Non-steroidal anti-inflammatory drugs, used to treat pain in inflammatory conditions such as arthritis, can affect renal function and therefore the effectiveness of antihypertensive medications [36, 37]. This highlights the importance of treating the complex combination of diseases present in individuals and not just individual diseases [38]. A comprehensive assessment of older persons rather than specialty specific assessments is most appropriate (i.e. comprehensive geriatric assessment [39]).

In all four countries there is socioeconomic disparity across disease patterns and also with respect to individual disease prevalences. The U.S. in particular have a much more pronounced socio-economic gradient than the other three countries for conditions such as lung disease, stroke, myocardial infarction, psychological illnesses and high blood pressure in adjusted models. Socioeconomic indicators for such as education and, particularly income, are also more pronounced for the U.S., Canada and England than for Ireland with respect to disease clusters. One possible reason may be that Ireland’s distribution of wealth is more equal than the other countries with the U.S. having the least equal distribution of the four countries. OECD estimates for the Gini coefficient: a measure of income equality shows that Ireland’s index was lower (more equal) than the U.S., Canada and England (0.308, 0.396, 0.320, 0.358 respectively for 2013). The work of Marmot [40] and Pickett and Wilkinson [41] for example, suggests that high levels of country-level inequality is harmful for population health, and some recent studies have documented interesting links between country-level inequality and inflammatory markers such as CRP [42]. The work of Zaninotto et al. [17] also noted that healthy life expectancy was significantly related to socioeconomic inequality and showed similar levels of disability-free life expectancy in the U.S. and England. Nevertheless, income inequality does not wholly explain the differences in disease prevalence and patterns, as the U.S. had consistently higher prevalence of most diseases at each level of the socioeconomic gradient when compared to other countries even after adjusting for confounding covariates.

The link between alcohol consumption and disease clusters across countries at first seems counterintuitive. For the U.S., Canada and England drinking alcohol was associated with reduced odds for at least two clusters whereas the opposite trend was found for Ireland. For the U.S., Canada and England the disease clusters negatively associated with alcohol consumption were those with the highest average number of medical conditions. Conversely, the two disease clusters positively associated with alcohol consumption for Ireland had the lowest mean number of morbidities. Therefore, the negative association for the U.S., Canada and England may be due to changes in behavior after disease diagnosis.

Excess weight represents a de-facto state of increased inflammatory signalling [43] which in turn increases risk for many chronic diseases [44]. Obesity is also a known risk factor for many of the conditions included in this study such as diabetes, hypertension, heart disease, osteoarthritis and certain types of cancer [45]. Having an elevated BMI increased the odds of being in three disease clusters across countries and had a pronounced effect on the individual prevalence of all conditions except osteoporosis and cancer. Self-reported obesity was highest for the U.S. (33.3%), similar for England and Canada (26.7, 26.2% respectively) and lowest for Ireland (24.2%). Although BMI was based on self-reported weight and height, our estimations are in line with WHO and other estimates for 2012 [46–49].

Two main limitations of this work are that, our analysis was limited to 10 chronic conditions and these conditions were self-reported doctor’s diagnoses. As such, prevalence of diseases may be underestimated in cases where a participant has not yet engaged with the healthcare system to get a diagnosis, or a participant may not report a chronic disease as the condition has been managed. To counteract the latter issue we included the lifetime prevalence of all conditions and so included participants as having a condition if they had ever reported disease incidence at any previous wave and had not later disputed it. Another limitation is that although risk factors such as BMI, smoking history, and alcohol consumption, were controlled for, the length of time being overweight/obese; alcohol/smoking intensity and duration and physical activity over the life course were not. The obesity epidemic started earlier in the U.S. across all age ranges. Childhood obesity in the U.S. in 1975 (relevant to the youngest participants) was more than double that of England, Canada and Ireland (5.5% U.S., 2.7% Canada, 2.7% UK, 1% Ireland) [50]. Adult obesity rates have increased dramatically in the last 45 years in all countries but have been persistently higher in the U.S.. Between 1975 and 2016, the obesity rate in the U.S. increased to 36.2% (from 11.6%) while in Canada it increased to 29.4% (from 9.8%), in the U.K. to 27.8% (from 9.4%) and in Ireland to 25.3% (from 6.4%) [51]. For eight of the 10 conditions studied, the U.S. had the highest overall prevalence and for six conditions Ireland has the lowest prevalence with respect to BMI. These trends align with the childhood and adult obesity rates from 1975 onwards. There is a large body of evidence to suggest that childhood habits and health factors are strong determinants of disease onset in adulthood [12, 52, 53] and although anecdotal it is possible that many of the adverse health patterns and disease patterns found may be due to the fact that the U.S. were the first to experience the obesity epidemic followed by Canada, England then Ireland.

## Conclusions

We have shown that the U.S. had significantly higher prevalence of multimorbidity and nearly all medical conditions studied compared to Canada, England and Ireland. This trend persisted even after controlling for age, sex, socio-economic and lifestyle factors.

The effect of socioeconomic status on disease patterns and individual disease prevalence was more pronounced in the U.S., Canada, and England, than for Ireland. This information can be used to better understand the complex nature of multimorbidity and identify appropriate prevention and management strategies for treating the unique disease patterns of multimorbidity in these respective countries.

The trends and patterns of disease prevalence across the four countries aligned closely with obesity trends since 1975, although anecdotal this may suggest that lifestyle habits and health behaviors over the life course may be likely drivers for the differences in later disease onset, multimorbidity and disease patterns.

## Supplementary Information

**Additional file 1.**

**Additional file 2.**

**Additional file 3.**

**Additional file 4.**

**Additional file 5.**

**Additional file 6.**

**Additional file 7.**

**Additional file 8.**

**Additional file 9.**

**Additional file 10.**

**Additional file 11.**

## Data Availability

TILDA data are available from the Irish Social Science Data Archive (www.ucd.ie/issda/), Gateway to Globing Aging (www.g2aging.org/), and Interuniversity Consortium for Political and Social Research (www.icpsr.umich.edu/icpsrweb/). The ELSA data were made available through the UK Data Archive. Instructions to access ELSA data can be found at https://www.elsa-project.ac.uk/accessing-elsa-data. Although all efforts are made to ensure the quality of the materials, neither the original data creators, depositors or copyright holders, the funders of the data collections, nor the UK Data Archive, nor the UK Data Service bear any responsibility for the accuracy or comprehensiveness of these materials. HRS data can be accessed from https://hrs.isr.umich.edu/data-products/access-to-public-data. CLSA does not currently have a publicly available version of their data however, access to CLSA and all studies can be provided upon request to the primary author B.H. email: HERNANDB@tcd.ie.
