# Supplementary material for: Comparisons of disease cluster patterns, prevalence and health factors in the USA, Canada, England and Ireland
Source: BMC Public Health. 2021 Sep 15;21:1674. doi: 10.1186/s12889-021-11706-8 (PMC8442402; doi:10.1186/s12889-021-11706-8)
Supplement: Supplementary file 1 — Additional file 1. [file 12889_2021_11706_MOESM1_ESM.docx]

**Additional File 1 Table A1 Health Care Expenditures, Mortality and Disability-Adjusted Life Years (DALY) for the U.S., Canada, England and Ireland in 2012***

|  | U.S. | Canada | England | Ireland |
| --- | --- | --- | --- | --- |
| Health Care Expenditures |  |  |  |  |
| Per capita total health care expenditure in current population (Value US dollars) | 8423.3 | 4285.7 | 2968.1 | 4373.3 |
| Mortality |  |  |  |  |
| All cause mortality per 100,000 population | 835.5 | 677.7 | 778 | 800 |
| Potential Years of Life Lost for Diseases: |  |  |  |  |
| Diabetes | 159.5 | 87.2 | 35.1 | 34.7 |
| Acute Myocardial Infarction | 204.6 | 150.8 | 158.9 | 152.5 |
| Circulatory System Diseases | 1160 | 591.7 | 727.1 | 728.8 |
| Respiratory System Diseases | 306.4 | 153.2 | 248.5 | 152.5 |
| Nervous System Diseases | 167.7 | 141 | 170 | 158.8 |

*Note. National Health Statistics reported are for the year 2012, or the nearest available equivalent year^13,14^
