# Supplementary material for: Comparisons of disease cluster patterns, prevalence and health factors in the USA, Canada, England and Ireland
Source: BMC Public Health. 2021 Sep 15;21:1674. doi: 10.1186/s12889-021-11706-8 (PMC8442402; doi:10.1186/s12889-021-11706-8)
Supplement: Supplementary file 2 — Additional file 2. [file 12889_2021_11706_MOESM2_ESM.docx]

Additional File 2 **Survey Design Methodologies and Covariates**

**1 Brief Overview of Survey Design Methodologies**

**U.S.: HRS Cohort**

The data from the U.S. were drawn from the 2012 wave 11 of the Health and Retirement Study (HRS) which is a population representative longitudinal study of older adults aged 50 and over that began in 1991. To be comparable to the other countries, the data for this study was taken from the 2012 wave 11 cohort of the HRS and included a total of n=10634 non-Hispanic whites.

The initial target population for wave 1 of the HRS included all adults in the contiguous United States, aged 51 - 61 (born during the years 1931 - 1941), who reside in households^3^. HRS respondents are given sample weights of zero (as an exclusionary measure) if they were living in a nursing home or an institutionalized residence. Household financial units were identified as the primary sampling units of this study and were selected according to a multi-stage sample whereby first stage sampling units were selected from the U.S. Metropolitan Statistical Areas and non-MSA counties with probability proportional to the size of the area. Within these areas a random sample of area segments (SSUs) were selected followed by a third level sampling stage of selecting household units within SSUs. Finally household financial units (the primary sampling unit of the HRS) were sampled within household units^3^. Samples have been replenished at various times since the inception of the HRS to maintain balance of the 51-61 subpopulation^4^.

The HRS oversamples African Americans and Latinos. Approximately 10% of interviews are done with proxy informants for HRS respondents who are unable, or unwilling to complete the survey, but are willing to have someone else (a spouse, or family member) do so on their behalf.

ELSA and TILDA the English and Irish Longitudinal Study in Ageing (respectively) were designed as sister studies to the HRS and much of their design, data collection and survey questions are harmonized to allow for cross country comparison.

**Ireland: TILDA Cohort**

A total of 6,924 participants were drawn from the 2012 wave 2 of the Irish Longitudinal Study on Ageing (TILDA), a population representative sample of predominately white individuals aged 50 and over from across the Republic of Ireland.

The target population for the TILDA survey is the population of persons aged 50 or over living in residential addresses in the Republic of Ireland, and their spouses or partners of any age. Samples were drawn from a sampling frame using the RANSAM system (based on the Irish Geodirectory). 3,115 geographical clusters into 3 strands of socio-economic status (high, medium and low) and the TILDA sample was based on a selection of 640 clusters, where probability of selection was proportional to the estimated population of each cluster. The second stage sampling procedure involved a selection of 50 households from within each of the 640 selected clusters. All people over 50 within a household were invited to participate as well as partners <50 of an eligible participant^5^. The TILDA study started in 2010 and selected participants were followed up every 2 years subsequently.

The TILDA study was approved by the Trinity College Faculty of Health Sciences Ethics Committee, and testing protocols conformed with the Declaration of Helsinki. All participants provided written, informed consent when they first participated in the study (at wave 1) and consent was repeated at wave 2 (the focus of this study), both written and verbal. Respondents in all cases were provided with copies of their signed consent forms.^5-6^

**England: ELSA Cohort**

The data from England were drawn from the 2012-2013 wave 6 of the English Longitudinal Study in Ageing (ELSA) which included a total of n=8,086 non-Hispanic whites which were the focus of this study. ELSA is a population representative longitudinal study of older adults aged 50 and over, living in private households in England and started in 2002 with data collection generally occurring every 2 years. ELSA eligibility criteria were different to those of the other studies and participants to wave 1 were recruited from those who took part in the Health Survey for England (HSE). The selected participants were choosing from participating households of the HSE where at least 1 person had agreed to follow-up and was born before 1 March 1952 (i.e. at least 50 at the start of the study)^8^. ELSA participants were provided sample weights of zero (as an exclusionary measure) if they were living in an institution or outside of England. As with TILDA partners of an eligible participant aged <50 were also invited to join the study. In a similar fashion to the HRS the sample was replenished at waves 3,4, and 6 to maintain age balance over time^8^.

**Canada: CLSA Cohort**

The CLSA is a population representative longitudinal study of the Canadian population. The data from Canada were drawn from the 2010-2015 baseline wave of the Canadian Longitudinal Study in Aging (CLSA) and included a total of n=37,949 non-Hispanic whites which were the focus of this study. The target population of the CLSA were community dwelling adults aged 45-85 from across Canada. There were two arms of the study: Tracking participants were interviewed by telephone (Computer Assisted Telephone Interview or CATI); Comprehensive participants were interviewed in person in their home (Computer Assisted Personal Interview or CAPI)and they attended a data collection site for physical and clinical assessments. A total of 21,241 people were randomly selected from across 10 Canadian provinces and submitted their data by telephone interview. A further 30,097 were randomly selected from areas which were geographically close (within 25-50km) to 11 data collection sites and provided data through home visits and by visiting the collection site in person^9^ . Those participants recruited to the CATI were taken from three sampling frames: The Canadian Community Health Survey on Healthy Aging; provincial health registries, and random-digit dialing (RDD). CAPI participants were recruited from using sampling frames from provincial health registries and random-digit dialing. For the Tracking arm, sampling was continued until a target of 20,000 CATI participants was achieved with a minimum of 125 participants in each age/sex category within the 10 Canadian Provinces where age was binned into 4 categories: 45-54, 55-64,65-74,75-85.

The CLSA study was approved by the 11 institutional Research Ethics Boards and all participants provided signed consent to participate.

**2 Information on Covariates used and harmonization of Self-Reported Disease Prevalence**

**Measurements of Disease Prevalence**

In all cases disease prevalence is measured as the self-reported life time prevalence of the disease in question and includes reported incidences of the condition from previous waves of each respective cohort study and corrects for later disputes of disease diagnosis.

**Covariates**

All models were controlled for age, sex, education, BMI, income and smoking history and employment status. This section will give information on how each of these were measured.

Age was measured in three categories 52-64,65-74,75-85. Income tertiles were used to define the “Low”, “Middle” and “High” categories for each country. Income tertiles for Ireland were: Low €0-19,999; Middle €20.000- €37,999; High €38000+. For England, income tertiles were: Low 0-17,015 GBP, Medium 17,016-29380 GBP; High 29,381 + GBP. For the United States, income tertiles were: Low $0-$33,609 USD; Medium $33,610-73,665 USD; High $73,666+ USD. Income tertiles for Canada were: $0-$49,999 CAD; $50,000-$99,999 CAD; $100,000 + CAD. Employment status was measured using the following four categorisations: Employed; Unemployed or performing unpaid work; Retired; work options are limited due to disability. Employment status was controlled for to ensure that income was reflective of socio economic status and was not just confounding for current income level due to employment status.

Smoking history was measured in three categories: Current, Former or Never. BMI was measured by calculated by dividing participants’ self-reported weight in kilograms by their height in metres-squared and was categorized as <25, 25-29 and ≥30.

In the U.S., education was separated into three groups: high school or less (typically 0-12 years), more than high-school but less than college (typically 13-15 years), college graduate or higher (typically ≥16 years). In the UK education level is defined less than “O-level” (typically 0-11 years), more than “O-level” up to and including “A-level” (typically 12-14 years), more than “A-level” (typically ≥14 years). In Ireland education level is defined as “Primary level or less” (typically 0-8 years); “Secondary level” (typically 13-14 years) and “Third level” which includes college graduate or higher (typically ≥14 years). In Canada, education is defined as “Grade 13 or lower” (typically 0-12) years, “Secondary School Graduate” (typically 13-15 years) and “Post-Secondary degree” (typically 16+ years).

Alcohol consumption was harmonized into the following categories: Respondent consumes alcohol less than once per year; more often than once per year but less than once per week; once per week; 2-3 times per week or 4-7 times per week.

**Harmonisation of Medical Conditions, Survey Questions and Variable Routing**

Here we will show the question number, wording and pseudocode explaining the routing for determining self-reported life time prevalence of each of the medical conditions included in this study. The aim of this to make replication and extension of our analysis easier and more transparent for other researchers.

The following are the list of conditions, variable numbers and questions for the HRS:

**Additional File 2 Table 1: Medical conditions, variable numbers and survey questions for HRS study**

| Condition | Variable | Survey Question |
| --- | --- | --- |
| High Blood Pressure | NC005  R11HIBPE | Has a doctor ever told you that you have high blood pressure or hypertension |
| Diabetes | NC010  R11DIABE | Has a doctor ever told you that you have diabetes or high blood sugar? |
| Cancer | NC018  R11CANCRE | Has a doctor ever told you that you have cancer or a malignant tumor, excluding minor skin cancer? |
| Myocardial Infarction | NC036, NC257,MC257,  LC040, KC040,  JC040, HC040, G1295, F1162, E834, W369, V407 | NC036 Has a doctor ever told you that you have had a heart attack, coronary heart disease, angina, congestive heart failure, or other heart problems?  NC257 Has a doctor ever told you that you have had a heart attack? |
| Angina | NC036,NC260,  MC260, LC045,  KC045, JC045, HC045, G1301, F1168, E840, W371, V409 | NC036 Has a doctor ever told you that you have had a heart attack, coronary heart disease, angina, congestive heart failure, or other heart problems? NC260 Has a doctor ever told you that you have angina? |
| Stroke | NC053  R11STROKE | Has a doctor ever told you that you have had a stroke? |
| Lung Disease | NC030  R11LUNGE | Has a doctor ever told you that you have chronic lung disease such as chronic bronchitis or emphysema? IWER: Do not include asthma |
| Arthritis | NC070  R11ARTHRE | Have you ever had, or has a doctor ever told you that you have arthritis or rheumatism? |
| Psych1 | NC065  R11PSYCHE | Have you ever had or has a doctor ever told you that you have any emotional, nervous, or psychiatric problems? |
| Osteoporosis | NC280 | Has a doctor ever told you that you have osteoporosis? |

The following are the list of conditions, variable numbers and questions for the CLSA:

**Additional File 2 Table 2: Medical conditions, variable numbers and survey questions for CLSA study**

| Condition | Variable | Survey Question |
| --- | --- | --- |
| High Blood Pressure | CCC_HBP | Has a doctor ever told you that you have high blood pressure or hypertension? |
| Diabetes | CCC_DIAB | Has a doctor ever told you that you have diabetes, borderline diabetes or that your blood sugar is high? |
| Cancer | Derived | What type of cancer were you disgnosed with:  Breast (1); Colorectal (2); Skin: melanoma (3); bladder (5); kidney (6); Lung (7); thyroid (8); Prostate (9); Ovarian (10); leukemia (11); Pancreatic (12); Non-Hodgkin lymphoma (13);Other (97) lip, oral cavity and pharynx cancer [_OR]; lip, oral cavity and pharynx cancer - ICD10 [_OR_SP]; Other digestive organs cancer [DG]; digestive organs cancer - ICD10 [DG_SP]; Other respiratory and intrathoracic organs cancer [_RS]; respiratory and intrathoracic organs cancer - ICD10 [RS_SP]; bone and articular cartilage cancer [_BN]; bone and articular cartilage cancer - ICD10 [_BN_SP]; Other female genital organs cancer [_FGO]; female genital organs cancer - ICD10 [_FGO_SP];Other male genital organs cancer [_MGO]; male genital organs cancer - ICD10 [_MGO_SP]; Other urinary tract cancer [_UR]; eye, brain & other parts of central nervous system cancer [_CNS]; eye, brain & other parts of central nervous system cancer - ICD10 [_CNS_SP]; endocrine glands (other than thyroid) cancer [EG]; ill-defined, secondary and unspecified sites cancer [_UN]; ill-defined, secondary and unspecified sites cancer - ICD10 [_UN_SP]; Other lymphoid, haematopoietic and related tissue cancer [_LHT]; lymphoid, haematopoietic and related tissue cancer - ICD10 [_LHT_SP], Do NOT include skin: non-melanoma (4), skin cancer: not specified [_SNS] |
| Myocardial Infarction | CCC_AMI | Has a doctor ever told you that you have had a heart attack or myocardial infarction? |
| Angina | CCC_ANGI | Has a doctor ever told you that you have angina (or chest pain due to heart disease)? |
| Stroke |  | Has a doctor ever told you that you have experienced a Stroke or CVA (cerebrovascular accident)? |
| Lung Disease | CCC_COPD | Has a doctor told you that you have/had any of the following: emphysema, chronic bronchitis, chronic obstructive pulmonary disease (COPD), or chronic changes in lungs due to smoking? |
| Arthritis | Derived | Has a doctor ever told you you have:  …osteoarthritis in the knee [CCC_OAKNEE] AND/OR….osteoarthritis in the hip [CCC-OAHIP] AND/OR …osteoarthritis in one or both hands [CCC_OAHAND] AND/OR ….other type of arthritis [combined CCC_ARTOT_COM; CCC_OTART_TRM] AND/OR rheumatoid arthristic [CCC_RA] |
| Psych2 | Derived | Has a doctor ever told you that you have.... an anxiety disorder such as a phobia, obsessive-compulsive disorder or a panic disorder [CCC_MOOD] AND/OR a mood disorder such as depression (including manic depression), bipolar disorder, mania, or dysthymia [CCC_ANXI] |
| Osteoporosis | CCC_OSTPO | Has a doctor ever told you that you have osteoporosis, sometimes called low bone mineral density, or thin, brittle or weak bones? |

The following are the list of conditions, variable numbers and questions for the ELSA:

**Additional File 2 Table 3: Medical conditions, variable numbers and survey questions for ELSA study**

| Condition | Variable | Survey Question |
| --- | --- | --- |
| High Blood Pressure | hedacbp hedanbp  hediabp  r6hibpe | hedacbp Previous Incidence: Our records show that when we last interviewed you you had high blood pressure or hypertension hedanbp Disputes: Code reason why respondent disputes having had HBP hediabp New Incidences: Apart from what you have already told us, and thinking about what has happened since we last saw [^you / [^name]] on [^date of last interview] has] a doctor [^BLANK / ever] told [^you / [^name]] that [^you have / he has / she has] (or have had) any of the [^BLANK / other] conditions on this card |
| Diabetes | hedacdi hedandi  hediadi  r6diabe | ^Diabetes or high blood sugar |
| Cancer | hedbdca hedbmca hedibca  r6cancre | hedbdca Previous Incidence: Our records show that when we last interviewed you you had cancer hedbmca Disputes: Code reason why respondent disputes having had cancer hedibca New Incidences: Apart from what you have already told us, and thinking about what has happened since we last saw [^you / [^name]] on [^date of last interview] has] a doctor [^BLANK / ever] told [^you / [^name]] that [^you have / he has / she has] (or have had) any of the [^BLANK / other] conditions on this card |
| Myocardial Infarction | HEDACMI HEDANMI,HEDIAMI  hedia01…10,heaga,  heagb, HeDiaC3, hedacmi, hedimmi | (wording A heart attack (including myocardial infarction or coronary thrombosis) |
| Angina | hedacan, hedanan, hediaan,  hedia01…10,heaga,  HeDiaC2,hedacan, hediman | (condition wording ANGINA) |
| Stroke | hedacst hedanst hediast  r6stroke | wording A stroke (cerebral vascular disease) |
| Lung Disease | hedbdlu hedbmlu hediblu  r6lunge | ^Chronic lung disease such as chronic bronchitis or emphysema |
| Arthritis | hedbdar hedbmar hedibar  r6arthre | ^Arthritis (including osteoarthritis , or rheumatism) |
| Psych1 | hedbdps hedbmps hedibps  r6psyche | ^Any emotional, nervous or psychiatric problems |
| Psych2 | hepsy1… hepsy9  hepsyde, hepsyan,  hepsyma | ^depression, manic depression or anxiety |
| Osteoporosis | hedbdos hedbmos ,hedibos,  hedib01… hedib10,  HeDiaD4, | ^Osteoporosis, sometimes called thin or brittle bones |

The following are the list of conditions, variable numbers and questions for the TILDA:

**Additional File 2 Table 4: Medical conditions, variable numbers and survey questions for TILDA study**

| Condition | Variable | Survey Question |
| --- | --- | --- |
| High Blood Pressure | ph201_01 | has a doctor ever told [you/Rname] that [you/he/she] [have/has] any of the conditions on this card?: High blood pressure or hypertension |
| Diabetes | ph201_05 | has a doctor ever told [you/Rname] that [you/he/she] [have/has] any of the conditions on this card?: Diabetes or high blood sugar |
| Cancer | ph301_05 | has a doctor ever told [you/Rname] that [you/he/she] [have/has] any of the conditions on this card?: Cancer or a malignant tumour (including leukaemia or lymphoma but excluding minor skin cancers) |
| Myocardial Infarction | ph201_03 | has a doctor ever told [you/Rname] that [you/he/she] [have/has] any of the conditions on this card?: A heart attack (including myocardial infarction or coronary thrombosis) |
| Angina | ph201_02 | has a doctor ever told [you/Rname] that [you/he/she] [have/has] any of the conditions on this card?: Angina |
| Stroke | ph201_06 | has a doctor ever told [you/Rname] that [you/he/she] [have/has] any of the conditions on this card?: A stroke (cerebral vascular disease) |
| Lung Disease | ph301_01 | has a doctor ever told [you/Rname] that [you/he/she] [have/has] any of the conditions on this card?: Chronic lung disease such as chronic bronchitis or emphysema |
| Arthritis | Ph301_03 | has a doctor ever told [you/Rname] that [you/he/she] [have/has] any of the conditions on this card?: Arthritis (including osteoarthritis, or rheumatism) |
| Psychological | Ph301_07 | has a doctor ever told [you/Rname] that [you/he/she] [have/has] any of the conditions on this card?: Any emotional, nervous or psychiatric problems,  such as depression or anxiety |
| Mood/Anxiety | ph316_02 ph316_03 ph316_08 | Please look at card PH7. What type of emotional, nervous or psychiatric problems [do/does] [you/he/she] have? ph316_02 Anxiety ph316_03 Depression ph316_08 Manic Depression |
| Osteoporosis | ph_301_03 | has a doctor ever told [you/Rname] that [you/he/she] [have/has] any of the conditions on this card?: osteoporosis |
