# Supplementary material for: Comparisons of disease cluster patterns, prevalence and health factors in the USA, Canada, England and Ireland
Source: BMC Public Health. 2021 Sep 15;21:1674. doi: 10.1186/s12889-021-11706-8 (PMC8442402; doi:10.1186/s12889-021-11706-8)
Supplement: Supplementary file 3 — Additional file 3. [file 12889_2021_11706_MOESM3_ESM.docx]

**Additional File 3 Cohort Characteristics**

**Additional File 3 Table 1: U.S. Cohort Characteristics: HRS wave 11, 2012 n (%)**

| Variable | Men  n =4824  (47.2%) | Women  n =6034  (52.8%) | Total  n =10858 |
| --- | --- | --- | --- |
| Age |  |  |  |
| 52 - 64 | 1961 (55.38) | 2352 (52.7) | 4313 (53.96) |
| 65 - 74 | 1506 (28.58) | 1981 (28.51) | 3487 (28.54) |
| 75 - 85 | 1357 (16.04) | 1701 (18.79) | 3058 (17.49) |
| Education Level |  |  |  |
| Low | 2059 (38.17) | 2961 (42.89) | 5020 (40.66) |
| Medium | 1151 (25.05) | 1573 (27.11) | 2724 (26.14) |
| High | 1614 (36.78) | 1500 (29.99) | 3114 (33.2) |
| Smoking Status |  |  |  |
| Never | 1685 (37.64) | 2952 (49.12) | 4637 (43.7) |
| Former | 2493 (47.61) | 2313 (37.61) | 4806 (42.33) |
| Current | 646 (14.75) | 769 (13.27) | 1415 (13.97) |
| BMI |  |  |  |
| <25 | 1106 (21.74) | 2151 (34.95) | 3257 (28.72) |
| 25-29.9 | 2171 (45.42) | 1898 (31.29) | 4069 (37.96) |
| ≥30 | 1547 (32.84) | 1985 (33.76) | 3532 (33.32) |
| Employment Status |  |  |  |
| Paid Employment | 1632 (43.88) | 1608 (35.6) | 3240 (39.51) |
| Unpaid/Unemployed | 133 (3.42) | 441 (7.71) | 574 (5.69) |
| Retired | 3008 (51.56) | 3890 (54.86) | 6898 (53.3) |
| Disabled | 51 (1.14) | 95 (1.83) | 146 (1.5) |
| Alcohol |  |  |  |
| < 1 per year | 1742 (31.85) | 2779 (41.66) | 4521 (37.03) |
| <1 per week | 691 (15.52) | 1067 (18.08) | 1758 (16.87) |
| 1 time per week | 1079 (24.03) | 1193 (22.27) | 1433 (14.73) |
| 2-3 times per week | 525 (12.34) | 423 (8.01) | 1461 (14.81) |
| 4-7 times per week | 787 (16.28) | 572 (9.98) | 1685 (16.57) |

**Additional File 3 Table 2. Canada Cohort Characteristics: CLSA baseline, 2010-2015 n (%)**

| Variable | Men  n =18961  (49.36%) | Women  n =18988 (50.64%) | Total  n =37949 |
| --- | --- | --- | --- |
| Age |  |  |  |
| 52 - 64 | 9286 (60.77) | 9630 (60.27) | 18916 (60.52) |
| 65 - 74 | 5240 (25.08) | 4948 (24.21) | 10188 (24.65) |
| 75 - 85 | 4015 (14.14) | 3528 (15.52) | 7543 (14.83) |
| Education Level |  |  |  |
| Low | 3308 (19.85) | 3654 (22.97) | 6962 (21.42) |
| Medium | 6451 (36.44) | 7185 (40.4) | 13600 (38.43) |
| High | 8818 (43.71) | 7267 (36.63) | 16085 (40.14) |
| Smoking Status |  |  |  |
| Never | 4253 (23.43) | 5874 (32.75) | 10127 (28.12) |
| Former | 12661 (66.38) | 10616 (57.39) | 23277 (61.85) |
| Current | 1627 (10.19) | 1616 (9.86) | 3243 (10.02) |
| BMI |  |  |  |
| <25 | 4539 (25.02) | 6563 (38.85) | 11102 (31.98) |
| 25-29.9 | 8885 (49.31) | 6330 (34.49) | 15215 (41.85) |
| ≥30 | 5117 (25.67) | 5213 (26.65) | 10330 (26.17) |
| Employment Status |  |  |  |
| Paid Employment | 6025 (38.74) | 5319 (33.18) | 11344 (35.94) |
| Unpaid/Unemployed | 232 (1.59) | 462 (2.28) | 694 (1.94) |
| Retired | 12062 (58.45) | 12021 (63.04) | 24083 (60.76) |
| Disabled | 222 (1.21) | 304 (1.51) | 526 (1.36) |
| Alcohol |  |  |  |
| < 1 per year | 2111 (10.8) | 2340 (11.71) | 4451 (11.26) |
| <1 per week | 4347 (23.1) | 6736 (37.19) | 11083 (30.2) |
| 1 time per week | 2006 (11.01) | 2002 (11.63) | 4008 (11.32) |
| 2-3 times per week | 4017 (22.91) | 3203 (18.27) | 7220 (20.58) |
| 4-7 times per week | 6060 (32.17) | 3825 (21.2) | 9885 (26.65) |

**Additional File 3 Table 3. England Cohort Characteristics: ELSA wave 2012-2013, n (%)**

| Variable | Men  n =3608 (47.75%) | Women  n =4478 (52.25%) | Total  n =8086 |
| --- | --- | --- | --- |
| Age |  |  |  |
| 52 - 64 | 1465 (52.16) | 1809 (48.69) | 3274 (50.35) |
| 65 - 74 | 1316 (30.27) | 1536 (30.16) | 2852 (30.21) |
| 75 - 85 | 772 (17.58) | 1040 (21.14) | 1812 (19.44) |
| Education Level |  |  |  |
| Low | 988 (28.9) | 1505 (35.66) | 2493 (32.43) |
| Medium | 900 (25.62) | 1356 (31.36) | 2256 (28.62) |
| High | 1502 (37.26) | 1173 (22.32) | 2675 (29.46) |
| Unknown | 163 (8.22) | 351 (10.67) | 514 (9.5) |
| Smoking Status |  |  |  |
| Never | 1059 (30.48) | 1839 (41.76) | 2898 (36.37) |
| Former | 2052 (54.77) | 1986 (43.44) | 4038 (48.86) |
| Current | 442 (14.75) | 560 (14.8) | 1002 (14.78) |
| BMI |  |  |  |
| <25 | 668 (18.78) | 1118 (25) | 1786 (22.03) |
| 25-29.9 | 1423 (39.2) | 1307 (29.32) | 2730 (34.04) |
| ≥30 | 874 (24.94) | 1230 (28.45) | 2104 (26.78) |
| Unknown | 588 (17.08) | 730 (17.23) | 1318 (17.16) |
| Employment Status |  |  |  |
| Paid Employment | 1153 (40.37) | 1083 (30.64) | 2236 (35.29) |
| Unpaid/Unemployed | 85 (3.7) | 368 (9.22) | 453 (6.38) |
| Retired | 2157 (50.98) | 2759 (55.24) | 4916 (53.21) |
| Disabled | 158 (5.38) | 175 (4.89) | 333 (5.13) |
| Alcohol |  |  |  |
| < 1 per year | 252 (6.99) | 603 (13.8) | 855 (10.54) |
| <1 per week | 504 (14.09) | 1027 (23.54) | 1531 (19.02) |
| 1 time per week | 744 (21.34) | 980 (22.67) | 849 (11.03) |
| 2-3 times per week | 664 (19.23) | 566 (12.78) | 1563 (19.88) |
| 4-7 times per week | 887 (22.95) | 659 (14.0) | 2088 (25.26) |
| Unknown | 502 (15.4) | 550 (13.21) | 1052 (14.26) |

**Additional File 3 Table 4. Ireland Cohort Characteristics****: TILDA wave 2 2012, n(%)**

| Variable | Men  n =3174  (51.07%) | Women  n =3750  (48.9%) | Total  n =6924 |
| --- | --- | --- | --- |
| Age |  |  |  |
| 52 - 64 | 1611 (57.86) | 1981 (58.28) | 3592 (58.06) |
| 65 - 74 | 972 (23.11) | 1063 (23.79) | 2035 (23.44) |
| 75 - 85 | 500 (19.03) | 541 (17.93) | 1041 (18.5) |
| Education Level |  |  |  |
| Low | 935 (29.49) | 867 (33.6) | 1802 (31.48) |
| Medium | 1210 (51.93) | 1471 (47.42) | 2681 (49.75) |
| High | 938 (18.58) | 1247 (18.98) | 2185 (18.78) |
| Smoking Status |  |  |  |
| Never | 1115 (36.23) | 1831 (49.56) | 2946 (42.62) |
| Former | 1486 (46.93) | 1197(32.24) | 2683 (39.82) |
| Current | 482 (16.84) | 557 (18.31) | 1039 (17.55) |
| BMI |  |  |  |
| <25 | 755 (24.33) | 1470 (39.33) | 2225 (31.59) |
| 25-29.9 | 1539 (49.84) | 1358 (38.25) | 2897 (44.23) |
| ≥30 | 789 (25.83) | 757 (22.41) | 1546 (24.18) |
| Employment Status |  |  |  |
| Paid Employment | 1194 (42.24) | 1096 (29.82) | 2290 (36.23) |
| Unpaid/Unemployed | 256 (9.61) | 1131(35.62) | 1387 (22.19) |
| Retired | 1497 (43.42) | 1227 (30.05) | 2724 (36.95) |
| Disabled | 136 (4.73) | 131 (4.51) | 267 (4.62) |
| Alcohol |  |  |  |
| < 1 per year | 408 (13.46) | 610 (17.95) | 1018 (15.63) |
| <1 per week | 509 (16.71) | 1033 (28.56) | 1542 (22.45) |
| 1 time per week | 501 (17.26) | 570 (16.28) | 1071 (16.79) |
| 2-3 times per week | 650 (20.74) | 511 (13.26) | 1161 (17.12) |
| 4-7 times per week | 483 (13.82) | 333 (7.91) | 816 (10.96) |
| Unknown | 532 (18.01) | 528 (16.03) | 1060 (17.05) |
