# Supplementary material for: Comparisons of disease cluster patterns, prevalence and health factors in the USA, Canada, England and Ireland
Source: BMC Public Health. 2021 Sep 15;21:1674. doi: 10.1186/s12889-021-11706-8 (PMC8442402; doi:10.1186/s12889-021-11706-8)
Supplement: Supplementary file 4 — Additional file 4. [file 12889_2021_11706_MOESM4_ESM.docx]

Additional File 4 Code

###############################################################################################

# Run the LCA analysis in R #

###############################################################################################

#first choose the number of groups...here we will use an approximation to the BIC using the formula

#log(sum(weights))*k - 2*log(quasilikelihood)

#k is the number of parameters estimated by the model...here it will be #groups + #params in regression

#i.e. intercept, age, sex, income, education, smoking history

#this script will run weighted LCA and also LCA regression.

#############################################################################################

# Choose Number of Groups #

#############################################################################################

library(lcca)

tot_groups<-10

#numparams<-6

log_lik<-rep(NA,tot_groups)

set.seed(100)

for(i in 2:tot_groups){

print(i)

lca.lcca<-lcca::lca(

cbind(HB, DIABETES, CANCER, STROKE, ANGINA, HEARTATTACK, PSYCH1, ARTHRITIS, LUNGDIS, OSTEOPOROSIS)~1,

nclass=i,constrain.rhos=FALSE,

data=CAPIsubset,iter.max=40000,flatten.gammas=F,

flatten.rhos=1,weights=weight_W2_capi_edu, clusters =as.factor(cluster) ,strata =stratum)

k=(i*M+i-1)

log_lik[i]<- log(sum(CAPIsubset$weight_W2_capi_edu))*k-2*lca.lcca$loglik.final

}

#plot the penalised log likelihood to choose the number of groups (wherever the elbow is higher =better)

plot(c(2:tot_groups),log_lik[-1],type="l",pch=1)

library(ggplot2)

lik_plot<-data.frame(cbind(GrNum=c(2:tot_groups),Est_BIC=log_lik[-1]))

jpeg("TILDA_BIC_9Conditions.jpeg", width = 7, height = 4, units = 'in', res = 600)

ggplot(lik_plot, aes(x = GrNum, y = Est_BIC))+ geom_line() +theme_classic()+ggtitle("LCA Estimated BIC: Ireland ")+xlab("Number of Groups")+ylab("Estimated BIC")

dev.off()

###############################################################################################

#############################################################################################

# Run Final Analysis: #

#############################################################################################

#for tilda data shows that 4 classes is optimal

#Re run the code with your chosen number of classes (for TILDA nclass=4 change as necessary)

set.seed(100)

lca.lcca2<-lcca::lcacov(

cbind(HB, DIABETES, CANCER, STROKE, ANGINA, HEARTATTACK, PSYCH1, ARTHRITIS, LUNGDIS, OSTEOPOROSIS)~ragender+age+EDU_CAT+hh_inc_tertile+BEHsmoke+BMI_CAT+ALCOHOL+EMPL,

nclass=5,constrain.rhos=FALSE,constrain.alphas=FALSE,stabilize.alphas=1,reference = 5,

data=CAPIsubset,iter.max=50000,

flatten.rhos=1,weights=weight_W2_capi_edu, clusters =as.factor(cluster) ,strata =stratum)

summary(lca.lcca2)

#assign observations to groups:

table(apply(lca.lcca2$post.probs,1,which.max))

| **Number of Groups** | **IRELAND** | **ENGLAND** | **CANADA** | **USA** |
| --- | --- | --- | --- | --- |
| **2** | 45334.94 | 51265.01 | 251499.2 | 88574.47 |
| **3** | 45107.26 | 51081.54 | 250407.9 | 88040.97 |
| **4** | 44966.14 | 50957.79 | 249742.5 | 87697.38 |
| **5** | 44931.55 | 50922.33 | 249633.0 | 87626.02 |
| **6** | 44923.72 | 50918.28 | 249576.5 | 87595.56 |
| **7** | 44920.30 | 50905.01 | 249520.5 | 87549.43 |
| **8** | 44915.42 | 50891.71 | 249504.9 | 87536.35 |
| **9** | 44915.70 | 50904.09 | 249501.5 | 87522.41 |
| **10** | 44934.27 | 50886.98 | 249487.8 | 87528.86 |

## Bayesian Information Criterion:

**Additional File 4 Table 1 Quasi Bayesian Information Criterion for 2-10 groups across each country.**

Although subjective, in all cases 4 clusters appeared to be the best compromise between biologically interpretable results, model parsimony and model fit as little improvement in the BIC was observed beyond this point.
