# Supplementary figures and images for: Comparisons of disease cluster patterns, prevalence and health factors in the USA, Canada, England and Ireland
Source: BMC Public Health. 2021 Sep 15;21:1674. doi: 10.1186/s12889-021-11706-8 (PMC8442402; doi:10.1186/s12889-021-11706-8)

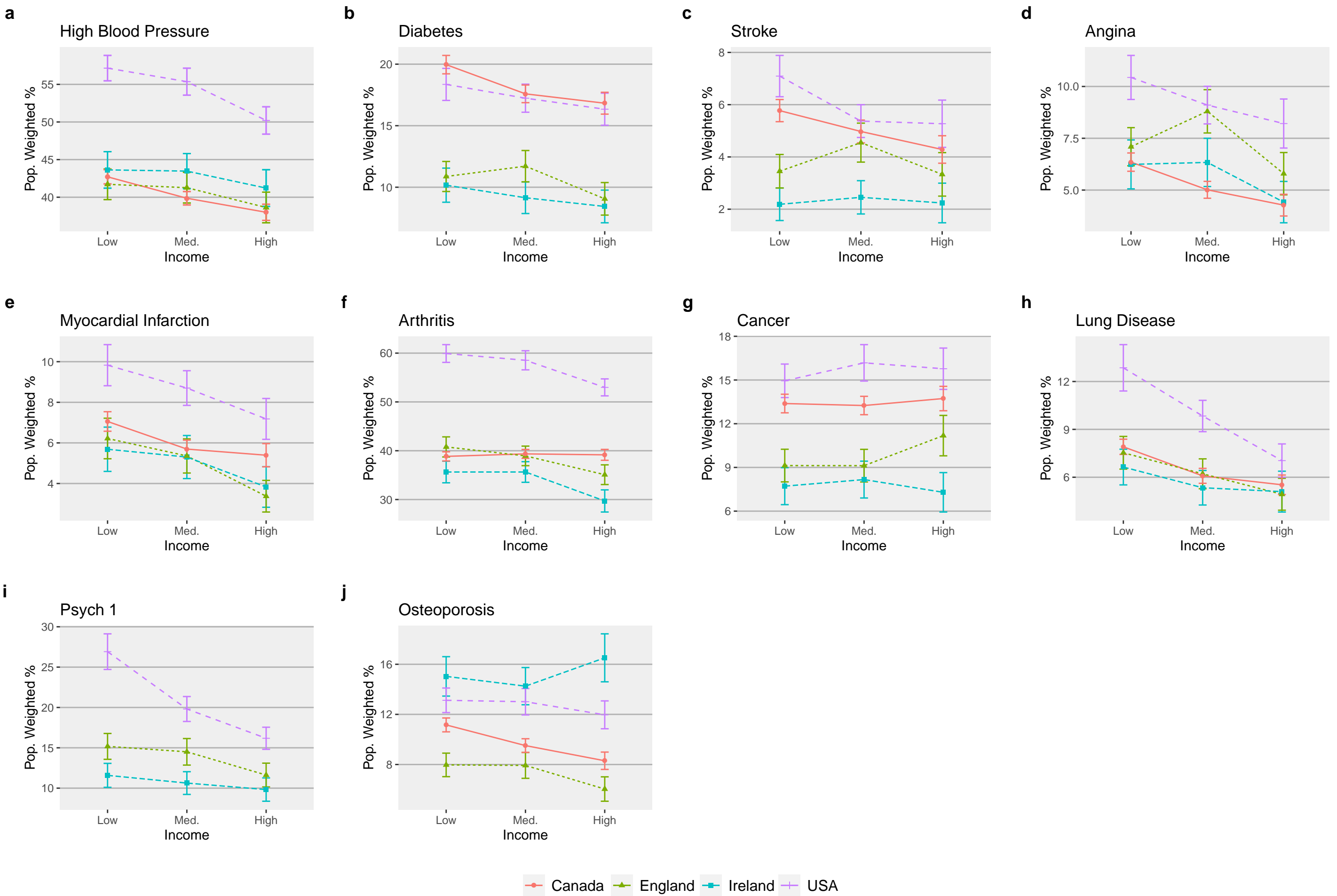

Supplement: Supplementary file 5 — Additional file 5. [file 12889_2021_11706_MOESM5_ESM.pdf]

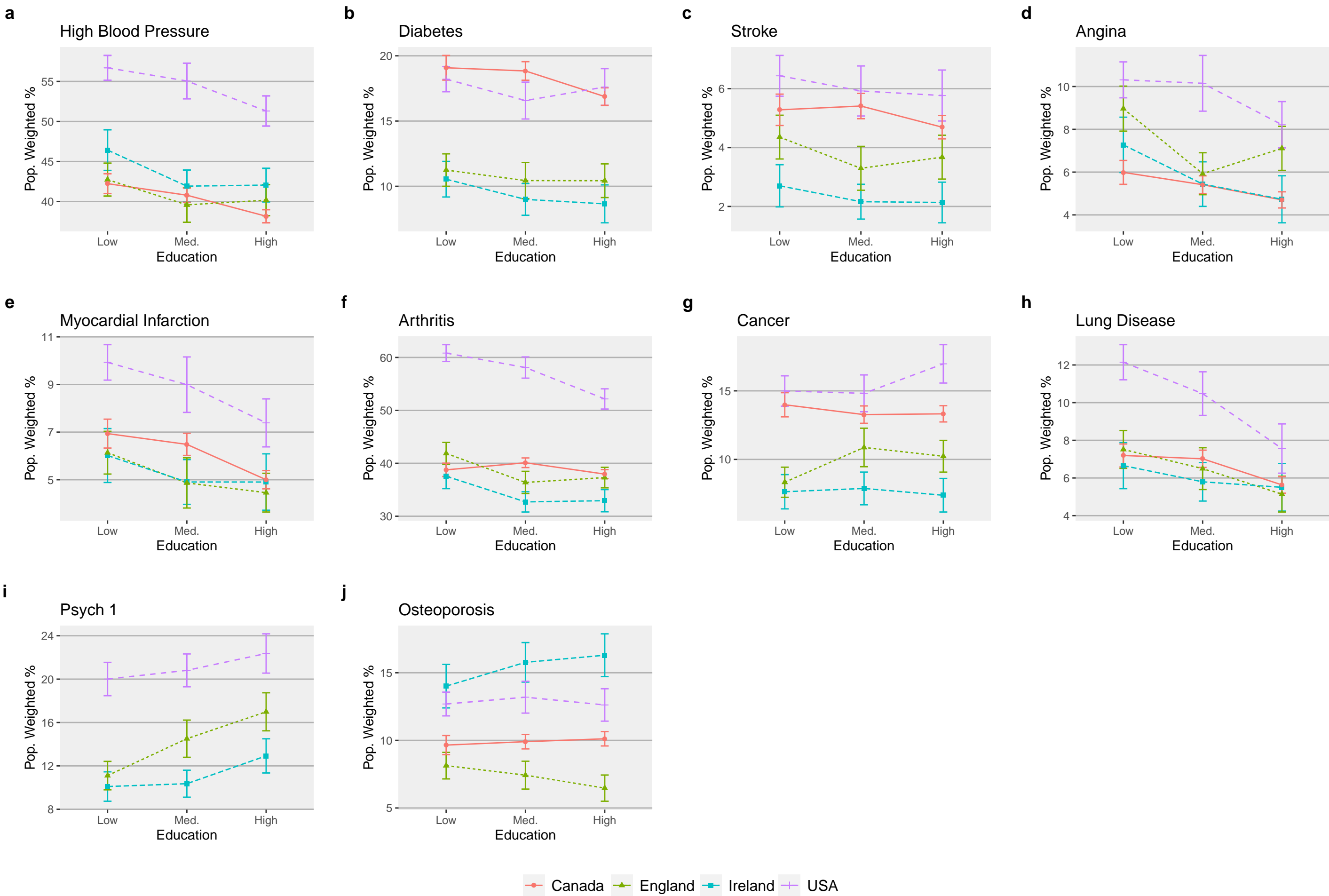

Supplement: Supplementary file 6 — Additional file 6. [file 12889_2021_11706_MOESM6_ESM.pdf]

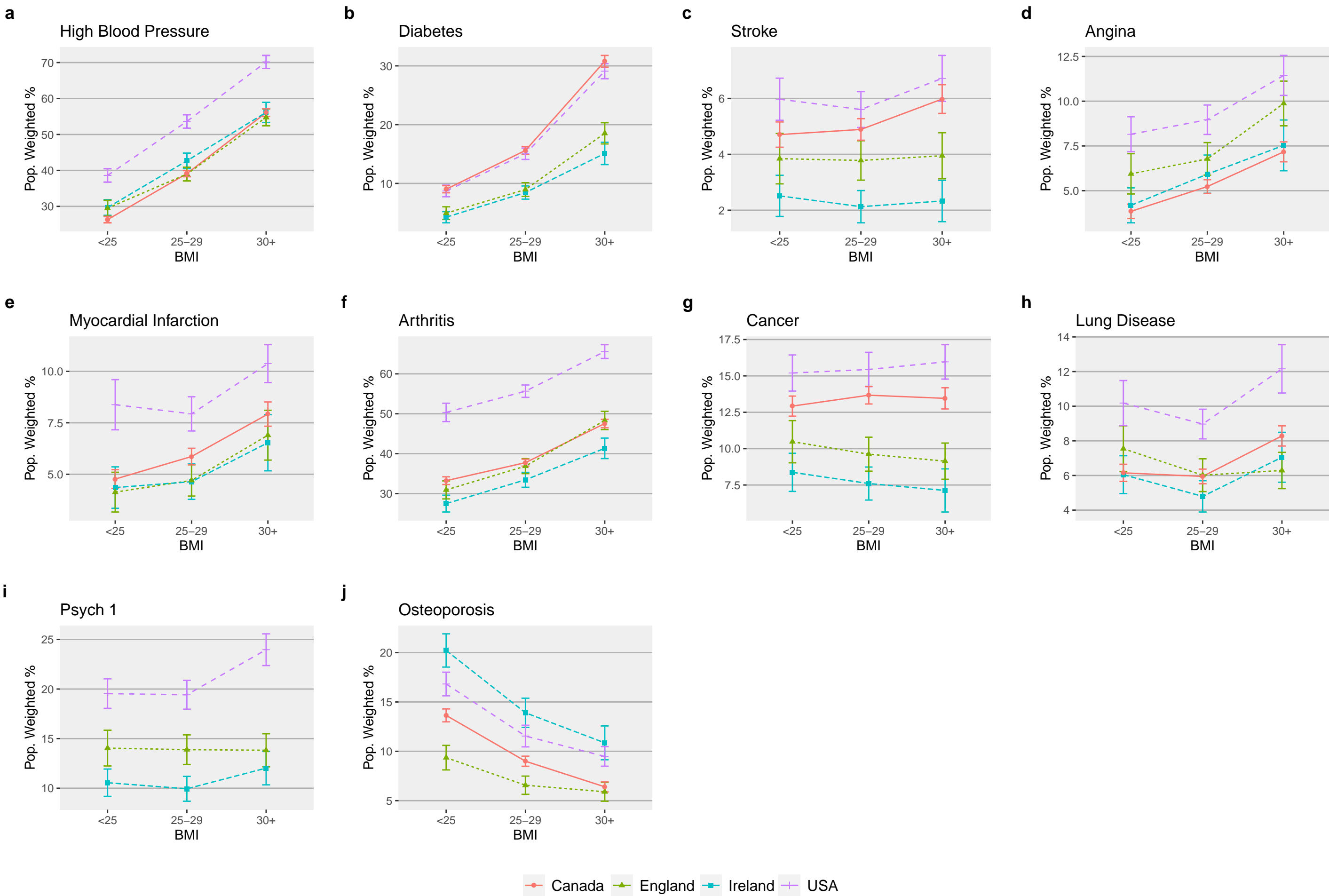

Supplement: Supplementary file 7 — Additional file 7. [file 12889_2021_11706_MOESM7_ESM.pdf]

# Canada Breakdown of Disease Patterns by Risk Factors

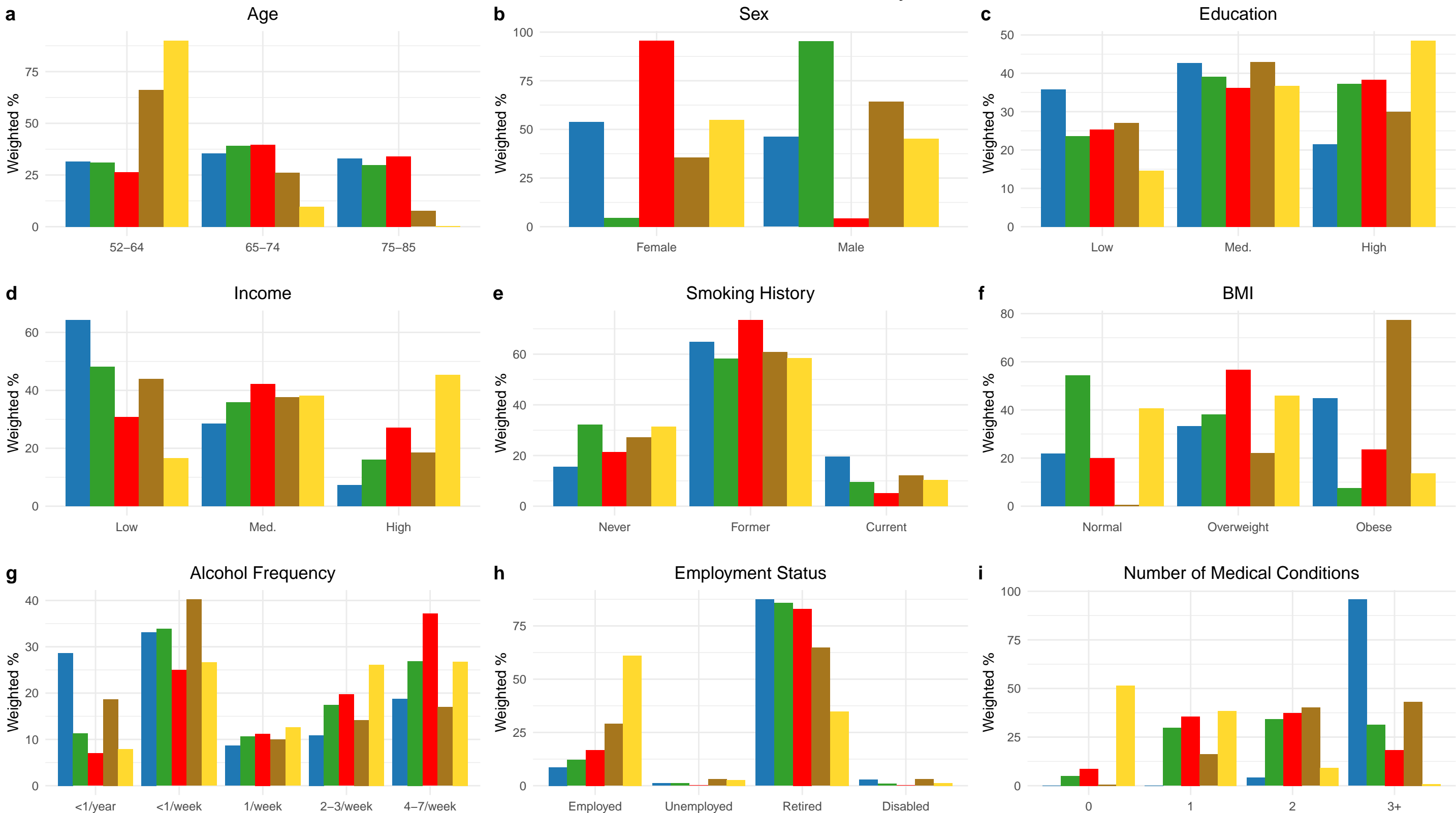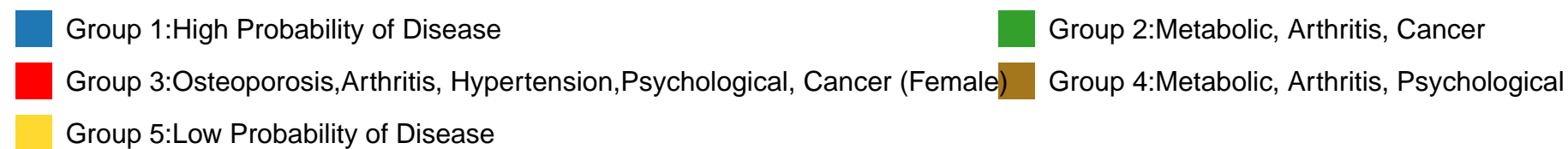

Supplement: Supplementary file 9 — Additional file 9. [file 12889_2021_11706_MOESM9_ESM.pdf]

# England Breakdown of Disease Patterns by Risk Factors

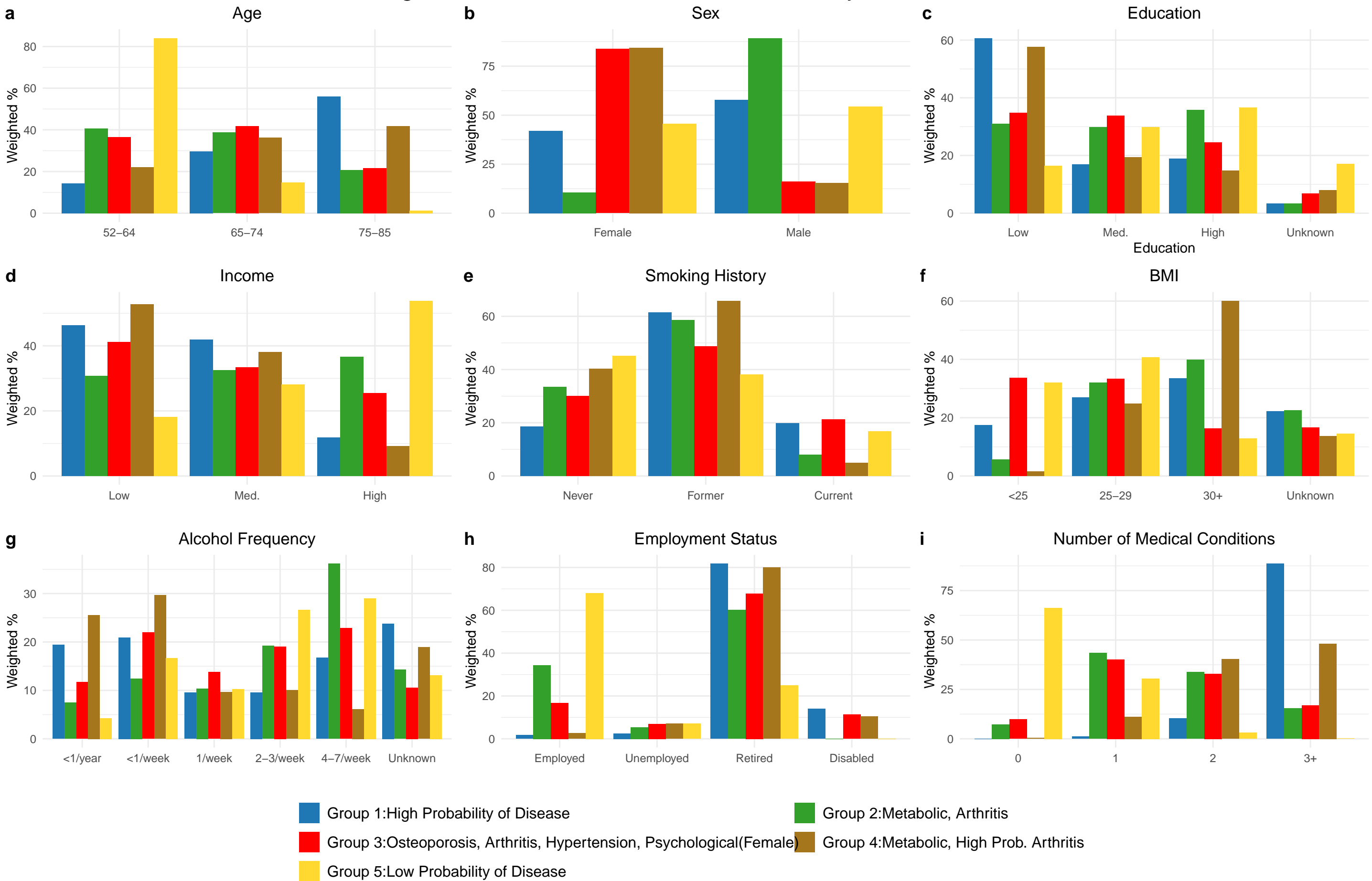

Supplement: Supplementary file 10 — Additional file 10. [file 12889_2021_11706_MOESM10_ESM.pdf]
