# Supplementary material for: Comparisons of disease cluster patterns, prevalence and health factors in the USA, Canada, England and Ireland
Source: BMC Public Health. 2021 Sep 15;21:1674. doi: 10.1186/s12889-021-11706-8 (PMC8442402; doi:10.1186/s12889-021-11706-8)
Supplement: Supplementary file 8 — Additional file 8. [file 12889_2021_11706_MOESM8_ESM.pdf]

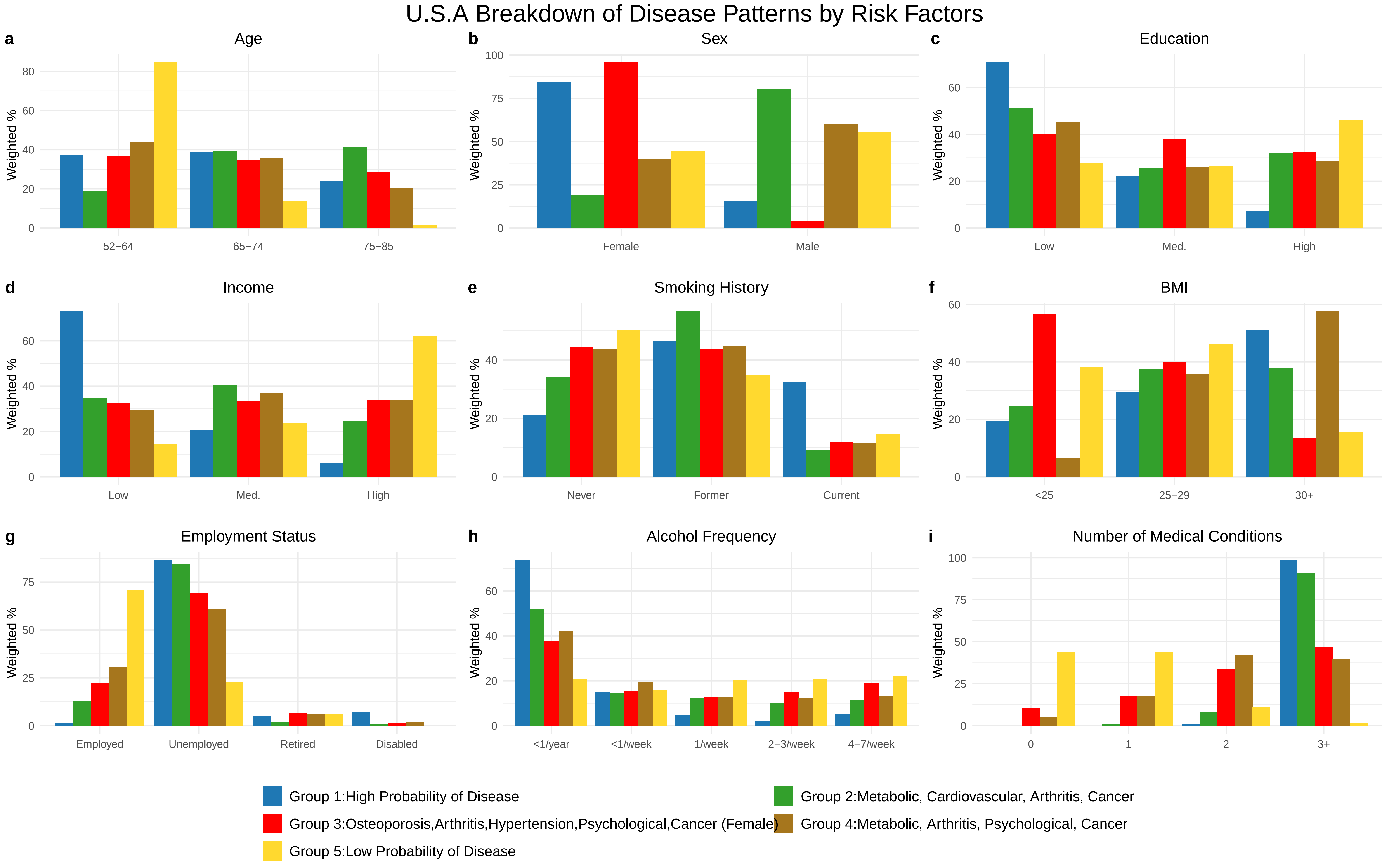
U.S.A Breakdown of Disease Patterns by Risk Factors
a
Age
b
Sex
c
Education
d
Income
e
Smoking History
f
BMI
g
Employment Status
h
Alcohol Frequency
i
Number of Medical Conditions
Weighted %
52−64
65−74
75−85
Female
Male
Low
Med.
High
Never
Former
Current
<25
25−29
30+
Employed
Unemployed
Retired
Disabled
<1/year
<1/week
1/week
2−3/week
4−7/week
0
1
2
3+
Group 1:High Probability of Disease
Group 2:Metabolic, Cardiovascular, Arthritis, Cancer
Group 3:Osteoporosis,Arthritis,Hypertension,Psychological,Cancer (Female)
Group 4:Metabolic, Arthritis, Psychological, Cancer
Group 5:Low Probability of Disease
